# Clinical applications of antimicrobial photodynamic therapy in dentistry

**DOI:** 10.3389/fmicb.2022.1020995

**Published:** 2023-01-05

**Authors:** Leila Gholami, Shiva Shahabi, Marzieh Jazaeri, Mahdi Hadilou, Reza Fekrazad

**Affiliations:** ^1^Department of Oral Biological and Medical Sciences, Faculty of Dentistry, University of British Columbia, Vancouver, BC, Canada; ^2^Dental Implants Research Center, Hamadan University of Medical Sciences, Hamadan, Iran; ^3^Dental Research Center, Hamadan University of Medical Sciences, Hamadan, Iran; ^4^Faculty of Dentistry, Tabriz University of Medical Sciences, Tabriz, Iran; ^5^Radiation Sciences Research Center, Laser Research Center in Medical Sciences, AJA University of Medical Sciences, Tehran, Iran; ^6^International Network for Photo Medicine and Photo Dynamic Therapy (INPMPDT), Universal Scientific Education and Research Network (USERN), Tehran, Iran

**Keywords:** antimicrobial photodynamic therapy, photo disinfection, photo activated disinfection, photodynamic therapy, caries, dentistry, periodontitis

## Abstract

Given the emergence of resistant bacterial strains and novel microorganisms that globally threaten human life, moving toward new treatment modalities for microbial infections has become a priority more than ever. Antimicrobial photodynamic therapy (aPDT) has been introduced as a promising and non-invasive local and adjuvant treatment in several oral infectious diseases. Its efficacy for elimination of bacterial, fungal, and viral infections and key pathogens such as *Streptococcus mutans*, *Porphyromonas gingivalis*, Candida albicans, and *Enterococcus faecalis* have been investigated by many invitro and clinical studies. Researchers have also investigated methods of increasing the efficacy of such treatment modalities by amazing developments in the production of natural, nano based, and targeted photosensitizers. As clinical studies have an important role in paving the way towards evidence-based applications in oral infection treatment by this method, the current review aimed to provide an overall view of potential clinical applications in this field and summarize the data of available randomized controlled clinical studies conducted on the applications of aPDT in dentistry and investigate its future horizons in the dental practice. Four databases including PubMed (Medline), Web of Science, Scopus and Embase were searched up to September 2022 to retrieve related clinical studies. There are several clinical studies reporting aPDT as an effective adjunctive treatment modality capable of reducing pathogenic bacterial loads in periodontal and peri-implant, and persistent endodontic infections. Clinical evidence also reveals a therapeutic potential for aPDT in prevention and reduction of cariogenic organisms and treatment of infections with fungal or viral origins, however, the number of randomized clinical studies in these groups are much less. Altogether, various photosensitizers have been used and it is still not possible to recommend specific irradiation parameters due to heterogenicity among studies. Reaching effective clinical protocols and parameters of this treatment is difficult and requires further high quality randomized controlled trials focusing on specific PS and irradiation parameters that have shown to have clinical efficacy and are able to reduce pathogenic bacterial loads with sufficient follow-up periods.

## Introduction

Photodynamic therapy (PDT) as a non-invasive and outpatient therapeutic method is an amazing field of photonic-based treatments with various applications in the medical field. It has evolved through the years with developments in photosensitizers (PS) and more complex methodologies resulting in revolutionary treatment outcomes in areas such as cancer therapy (Shi et al., 2019), alleviation of autoimmune disease complications (Gallardo-Villagrán et al., 2019), wound healing improvement (Tardivo et al., 2014; Oyama et al., 2020), and control or elimination of viral, fungal and bacterial infections in both planktonic or biofilm forms (Hu et al., 2018).

In recent years and with the emergence of multi-drug resistant bacteria, antimicrobial photodynamic therapy (aPDT) has attracted more attention than ever in medicine (Chung and Toh, 2014; Hu et al., 2018).

The mechanism behind the occurrence of the desired photodynamic reaction relies on three components of light, a light sensitive agent/photosensitizer (PS) and ambient oxygen molecules. The PS irradiated with a compatible light wavelength converts from singlet base state to singlet exited state, then converts to base state by releasing absorbed energy or with the occurrence of an intersystem crossing, it goes through two types of reactive oxygen species (ROS) production pathways. Both pathways are oxygen dependent (Castano et al., 2005; Robertson et al., 2009). The first, is to transfer an exited electron to other substrates such as other molecules or cell structures resulting in free radicals which produce ROS by reacting with triplet state oxygen. The second pathway occurs by energy transfer directly from the PS to the base state triplet oxygen molecules to be converted to exited singlet state with extremely oxidating characteristics (Hu et al., 2018). These two pathways happen simultaneously and the dominancy of each depends on oxygen concentration, PS characteristics, pH and dielectric constant of tissue (Kwiatkowski et al., 2018).

**Figure 1:**
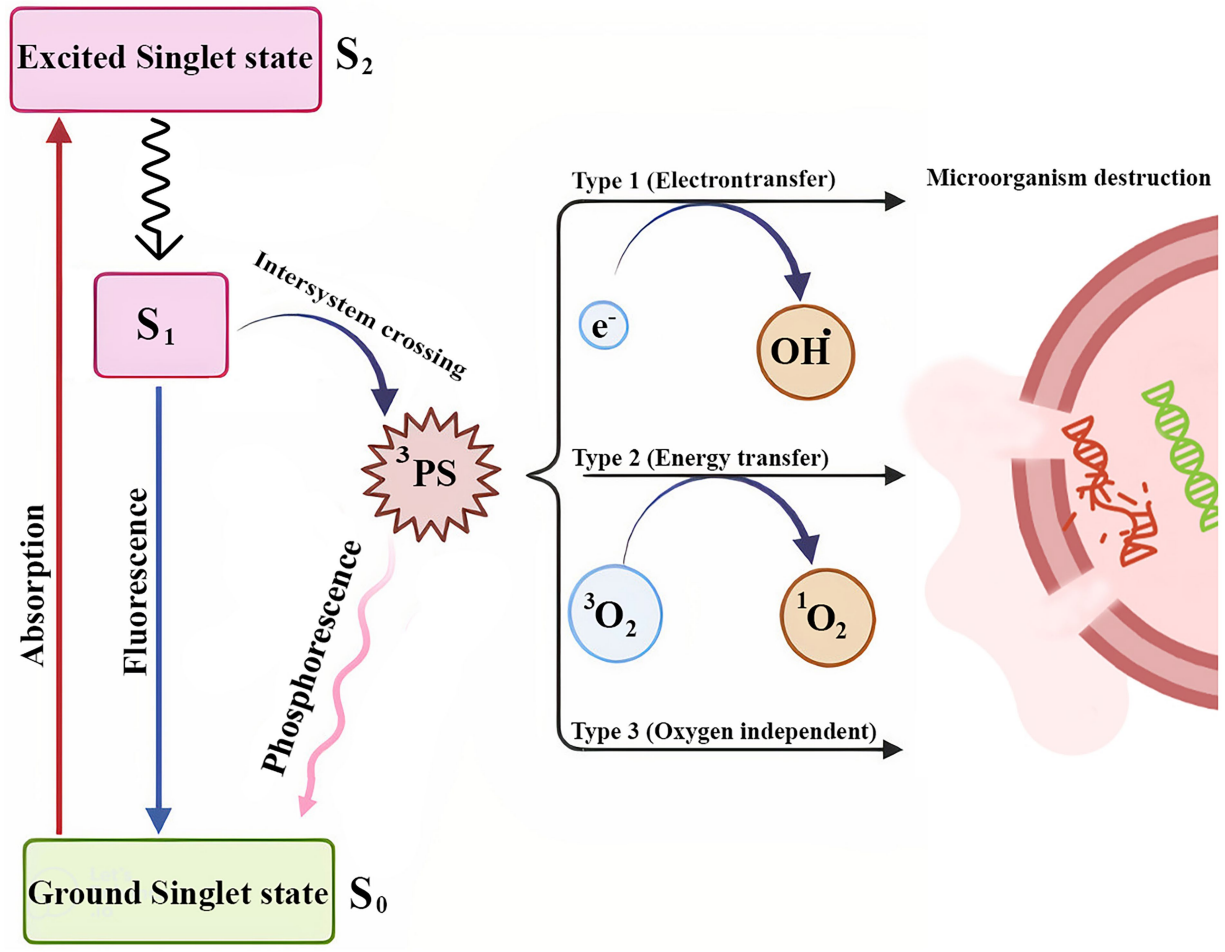
Schematic illustration of the three types of photochemical mechanisms involved in PDT.

Recently, a new mechanism has been purposed as Type III pathway, which is oxygen independent and not limited to the visible light spectrum. This mechanism may be found in anerobic/hypoxic conditions and with PSs such as psoralens and tetracyclines and with the addition of organic salts such as potassium iodide and sodium azide (Hamblin and Abrahamse, 2020). Due to the diverse intracellular metabolism pathways being hit by ROS agents, developing resistance against aPDT is highly unlikely (Azaripour et al., 2018), although, changes in virulence factor, adaptation or escape of microorganism from these light-based treatments need to be further explored (Marasini et al., 2021; Figure 1).

There are other methods such as phototherapy and photothermal therapy that need to be distinguished from photodynamic therapies (Ebrahimi et al., 2021; Zhao et al., 2021).

Photosensitizers can be activated by different light sources. Lasers are a group of light sources that irradiate high density light but currently considered as expensive devices (Juzeniene et al., 2004), on the other hand, light sources such as light emitting diodes (LED) are cheaper and more compact than traditional lasers (Rajesh et al., 2011). Broadband spectrum light sources such as lamps could also be used in PDT (Brancaleon and Moseley, 2002). Most of the common photosensitizers used in medicine are activated by the near red-light spectrum (630–700 nm; Salva, 2002; Ozog et al., 2016). High wavelengths lack the energy to excite the oxygen molecules and low wavelengths lack the ability to penetrate through the skin and reach to the target regions and may have the potential to induce photodamage to the tissues. Also, being respondent to low wavelengths leads to the activation of PS by sunlight which can interfere with the treatment. Furthermore, the PS’s activation spectrum should not overlap the body’s endogenous dyes’ absorbance range such as oxyhemoglobin, hemoglobin, and melanin (Allison et al., 2004; Kwiatkowski et al., 2018).

Safety and approval of photosensitizer molecules to be used *in vivo* is an important aspect to be considered. The eligibility of a PS to be administered for clinical use can be evaluated in various aspects: (1) Toxicity: PSs must not be toxic or generate toxic by-products during their metabolization in the body. (2) Elimination time: They must have an appropriate half-life and be removed from the body when they are not necessary. (3) Selectivity: They must accumulate only in the desired regions of the body intended to be treated and prefer to target micro-organisms or intended tumor cells instead of normal body cells. (4) Appropriate irradiation wavelength: They should respond to appropriate light wavelengths (in the optical window range 600–800 nm). (5) Easy clinical administration. (6) Biochemistry: Sufficient solubility in water to easily be transferred in the circulatory system. (7) Availability: Their production should be feasible in a commercial scale (Allison et al., 2004; Konopka and Goslinski, 2007; Sperandio et al., 2013; Kwiatkowski et al., 2018).

The available PSs in medicine have been evolved through time. Nowadays PSs can be divided into four groups as follows: (1) Tetrapyrrole structures, (2) Synthetic dyes, (3) Natural products, and (4) Modified PSs (Abrahamse and Hamblin, 2016). It is important to use approved photosensitizers *in vivo* and have sufficient invitro and preclinical studies to investigate their toxicity suitable clearance rate from tissues and determine safety for clinical applications in dentistry.

Some of the Ps considered non-toxic and approved for intraoral use are Methylene blue and toluidine blue-O which are approved phenothiazinium salts from the synthetic dyes group that have the most frequent application in dentistry and aPDT (Asnaashari et al., 2017; Okamoto et al., 2020; Ramalho et al., 2021; Romero et al., 2021) Their structural cationic charge facilitates an easy penetration to the gram-negative bacteria’s outer membrane with a high affinity to bacterial cells over body cells (Gomer, 1991). Other PSs that have been considered safe and used to eradicate microorganisms involved in oral and dental diseases are synthetic fluorescent dyes such as the Indocyanine green (Nikinmaa et al., 2021; Wadhwa et al., 2021), natural compounds such as Curcumin (Mendez et al., 2021; Moradi et al., 2021), nanosized natural zeolite (Ghazi et al., 2021), Riboflavin (Moradi et al., 2021), and Rose Bengal (Li et al., 2021). Various nanoparticle-integrated PSs have also been developed and investigated for their safety and efficacy in clinical applications (Hendi et al., 2021; Shahmoradi et al., 2022). There is a potential for side effects such as teeth staining, local damage to tissues and cells, possibility of overheating of the oral tissues and also risk of damage to eye and skin with overexposure to high intensity irradiations that should be considered when conducting clinical studies (Nielsen et al., 2015; Costa et al., 2016; Afrasiabi et al., 2022; Solarte et al., 2022).

Limiting factors in clinical applications of PDT such as the necessity of sufficient oxygen molecules has been an issue in the application of PDT in treating solid tumors or deep anerobic and hypoxic tissues such as deep periodontal pockets which has been the focus of researchers in recent years. In this regard, application of H_2_O_2_ (Yang et al., 2019) and oxygen self-sufficient nanoplatforms (Sun et al., 2021) in aPDT have shown promising results, since they increase the oxygen density in the target tissues which leads to more successful and functional aPDT.

Nano or porous structure incorporated PSs have also been a step forward in PS developments. Nanostructure incorporation of PS provides different functionalities and physicochemical properties such as magnetism and luminescence which is dependent to the type of the nanostructure. They also could extend the bioavailability of PSs by preventing rapid renal and hepatic clearance (Escudero et al., 2021). Moreover, the hydrophobic PSs’ solubility in water can be improved by encapsulating them (Alonso, 2004; Couvreur et al., 2006). Nanostructures may also enable drug release in desired tissues or times (Mura et al., 2013). Moreover, photoimmunotherapy (PIT) and monoclonal antibody technology has been used to improve the selectivity of the PSs (Schmitt and Juillerat-Jeanneret, 2012).

Overall, the broad spectrum of action and low level of adverse effects associated with PDT has made researchers eager to look to antimicrobial effects of PDT as suitable alternatives to conventional methods of treating infections. Microbial biofilms are responsible for almost 80% of human body infections led by bacteria or fungi (Høiby, 2017). The biofilm structure prevents the infiltration of immune system agents and antibiotics (Stewart, 2003) due to the altered physiological and metabolic properties of bacteria in biofilms compared to planktonic cells (Donlan, 2002). Therefore, microbes occupying a biofilm are able to tolerate 10–1,000 times greater antibiotic levels compared to planktonic ones (Ceri et al., 1999). The unique and local antimicrobial treatment method of aPDT is of great value in treating infections associated with microbial biofilms. Moreover, it has no undesirable systemic effects to the liver, kidney function and the commensal microbiota of the intestine. Recent findings on the existence of a gut–lung–brain axis and the role of gut microbiota in immunomodulation, and local and long-distance health and disease homeostasis adds to the importance of local antimicrobial treatment methods as alternative to systemic drugs (Liu et al., 2015; Enaud et al., 2020). Although aPDT has been reported to be effective against microbial biofilms, however, disruption oral bacterial biofilm in niches such as deep pockets prior to aPDT is of great importance and the treatment should be considered an adjunctive to conventional treatment of conditions such as periodontal and root canal infections for optimal results (Quishida et al., 2015; Cieplik et al., 2018; Hu et al., 2018).

A considerable number of studies have been conducted on the effects of PDT on microorganisms involved with oral diseases, including *Porphyromonas gingivalis* (*P. gingivalis*; Ding et al., 2021; Ghazi et al., 2021; Oruba et al., 2021; Yoshida et al., 2021) as a gram-negative obligate anaerobe bacterium (Hajishengallis et al., 2012) which is the main representative of pathogenic bacteria involved in periodontal diseases (Ding et al., 2021), *Streptococcus mutans* (*S. mutans*; Benine-Warlet et al., 2022; Dos Santos et al., 2022; Fernandes et al., 2022) which is the main bacteria involved in caries development, and Candida albicans which is the main opportunistic fungus in the oral microbial flora (de Lapena et al., 2022; Dos Santos et al., 2022).

In recent years several clinical randomized controlled trials have also been designed to evaluate the efficacy of this method in treating oral infectious diseases such as periodontitis (Wadhwa et al., 2021; Arsic et al., 2022), periimplantitis (Shatha-Subhi et al., 2022; Shetty et al., 2022), halitosis (Llanos do Vale et al., 2021; Romero et al., 2021), recurrent herpes labialis (Ramalho et al., 2021), root canal disinfection (Asnaashari et al., 2017; Okamoto et al., 2020), oral plaque and caries control (Nikinmaa et al., 2021), and treating fungal diseases caused by candida albicans (Alves et al., 2020).

With the potential of aPDT in eradicating microbial organisms and its rapid and daily development in recent years, this study aimed to review and provide an overall map of the available evidence in different clinical application areas of aPDT in dentistry.

## Search strategy

PubMed (Medline), Web of Science, Scopus and Embase databases were searched up to September 2022 to retrieve related clinical trials with the different combinations of manual keywords and the ones obtained from MeSH terms and Entree including “aPDT,” “antimicrobial photodynamic therapy,” “antibacterial photodynamic therapy,” “dent,” “oral,” “viral,” “bacteria,” “fungal,” “periodontitis.” Detailed search strategies in the databases are provided as Additional file 1. All reviews, *in-vitro* and animal studies were excluded, clinical studies were all investigated but only randomized clinical trials were included for data extraction. The studies resulted from the search were screened by the authors first based on the titles and abstracts, then based on the full texts. Finally, 89 RCT were identified and subclassified based on the type of infection they were focusing on (Additional file 1 attached). Six main oral infections/conditions were identified with randomized controlled clinical trials conducted on the application of aPDT for their treatment. The extracted data from final included papers included author name, publication year, study groups, investigated pathology, photosensitizer type/concentration, investigated microorganism, light type and irradiation parameters, follow-up periods, and the outcome of the individual studies.

## Antimicrobial photodynamic therapy and periodontal and peri-implant diseases

Periodontal disease is characterized by the progressive loss of alveolar bone and the supporting periodontium surrounding the teeth or dental implants (Papapanou et al., 2018; Tonetti et al., 2018). The etiology of this disease is widely varied and may have different origins, such as developmental, inflammatory, traumatic, neoplastic, genetic, or metabolic conditions (Pihlstrom et al., 2005; Schwarz et al., 2018). Pathologic microorganisms in the biofilm or dental plaque adjacent to the root surface and their interactions with the host immune response are the principal cause of periodontal diseases. The most popular microorganisms involved are *Aggregatibacter actinomycetemcomitans* (*A. actinomycetemcomitans*), *Fusobacterium* spp., *Porphyromonas gingivalis*, *Prevotella* spp., *Treponema denticola*, and *Streptococcus beta-hemolytic* (Papapanou, 2002; Theodoro et al., 2015). Thus, the main goal of periodontal therapy is to reduce or even eliminate the biofilm (Koyanagi et al., 2013).

Scaling and root planning (SRP) is the most common therapeutic method to achieve this goal. However, SRP has limitations regarding eradicating pathogens in cases where mechanical instruments have restricted access (Umeda et al., 2004). As a result, complementary treatments are needed to help eliminate periodontal pathogens. Local and systemic antibiotics have been proven effective in intra-oral biofilm control and adjunctive to periodontal treatment; however, their application is accompanied by unwanted side effects such as antibiotic resistance and gastrointestinal disorders (Feres et al., 2015; Carcuac et al., 2016). Therefore, studies have focused on methods such as aPDT as a non-invasive alternative treatment modality that does not result in side effects.

Pathogenic microorganisms are not the only predisposing factor for periodontal diseases. Several genetic and environmental factors have also been associated with periodontal diseases, including tobacco and alcohol usage, impaired host response, stress, nutrition, osteoporosis, and diabetes (Pihlstrom et al., 2005; Van Dyke and Sheilesh, 2005). Research shows that aPDT can be an effective adjunctive treatment in such populations with HIV-associated periodontitis, diabetic patients with periodontitis, smokers and patients undergoing orthodontic treatments with appliances that make plaque control difficult (Noro Filho et al., 2012; Theodoro et al., 2018; Alshahrani et al., 2020; Cláudio et al., 2021).

Applying aPDT has raised some concerns regarding potential side effects on host cells. However, according to the literature, the intensity of the light required for bacterial eradication is much lower than the toxic limit for host cells. Furthermore, the aPDT effects on host cells have benefited the treatment process. Some studies have indicated that aPDT can inactivate the inflammatory mediators such as tumor necrosis factor-α (TNF-α) and interleukin-1β (IL-1β) that diminish the periodontal restoration and, as a result, promote angiogenesis and restorative processes (Braham et al., 2009; Séguier et al., 2010).

Numerous studies have investigated the effect of aPDT on periodontal diseases that range from *in-vitro* studies, animal studies to RCTs and systematic reviews and meta-analyses. *In-vitro* studies have focused on the susceptibility of different periodontal disease-related microorganisms to aPDT. The application of aPDT has been shown to reduce several periodontal disease-associated bacteria such as *P. gingivalis, A. actinomycetemcomitans*, and *Fusobacterium nucleatum (F. nucleatum)* in biofilms (Prasanth et al., 2014; Kranz et al., 2015; Yoshida et al., 2017; Valle et al., 2019; Oruba et al., 2021). Kikuchi et al.’s review confirmed the susceptibility of bacteria to aPDT in different forms of planktonic cultures, plaque scrapings, and biofilms. They also reported the most common PSs used in periodontal therapy as follows: methylene blue, methylene blue-loaded polymeric nanoparticles, toluidine blue O, phthalocyanine, hematoporphyrin HCl, hematoporphyrin ester, chlorin e6, indocyanine green, indocyanine green-loaded nanospheres, and safranine (Kikuchi et al., 2015). The safety of most of these PS have to be considered *in vivo* applications. Although many studies have suggested that indocyanine green without laser irradiation is not significantly toxic to cells, it should be considered that under light activation, indocyanine green could act differently (Ateş et al., 2018). Researchers have found that the temperature rises as the laser irradiation time increases, leading to more significant cell toxicity. Moreover, the greater the concentration of indocyanine green, the higher its cytotoxicity effect (Solarte et al., 2022).

Systematic reviews have been conducted on the results of RCTs and the adjuvant effect of aPDT on periodontal diseases. Moro et al. included 22 RCTs in a systematic review comparing SRP alone and SRP associated with aPDT with at least 3-month follow-ups. According to their results, the association between SRP and aPDT depicted a significant clinical attachment level (CAL) gain and probing pocket depth (PPD) reduction. Indocyanine green as a PS and high concentrations of phenothiazine chloride showed clinical effectiveness in adjunctive aPDT treatment (Moro et al., 2021). Zhao et al. systematically reviewed the effect of aPDT versus antibiotics in treating periodontal diseases. Although there are controversial results, overall, they concluded that aPDT could replace antibiotics in treating both peri-implantitis and periodontitis (Du et al., 2021). In another systematic review, Chambrone et al. reviewed 26 RCTs and suggested that aPDT might yield clinical improvements in PD and CAL comparable to those resulting from conventional periodontal therapy for both periodontitis and periimplantitis (Chambrone et al., 2018). In another recent systematic review, the effect of aPDT on non-surgical management of untreated periodontitis cases the number of available studies were considered low with a high level of heterogeneity. A metanalysis was conducted to investigate changes in PPD, which failed to show any statistically significant difference (Salvi et al., 2020).

Future clinical studies need to be designed carefully to reduce the heterogenicity observed among current available literature and allow systematic reviews to be conducted investigating more specific clinical questions, narrowing down inclusion criteria and performing metanalysis of data to reach evidence-based conclusions.

## Antimicrobial photodynamic therapy in periodontitis

A considerable number of clinical studies have focused on evaluating the effect of aPDT in the treatment of various periodontal diseases, including previously termed aggressive and chronic periodontitis, necrotizing periodontitis, and the maintenance phase of periodontal therapy (Table 1). The main PSs used in the available literature were phenothiazine chloride, toluidine blue, methylene blue, and indocyanine green. Light sources with wavelengths in the range of 400–1,000 nm have been used with irradiation parameters that were different between studies, making it hard to suggest a suitable irradiation protocol.

**Table 1:** aPDT treatment in patients with periodontitis.

Author, year	Study design	Treatment groups	Investigated pathology	Photosensitizer, concentration	Light type and parameters (wavelength, power, power density, irradiation time) and frequency of irradiation	Microorganisms	Follow-up periods	Outcomes
Al-Momani (2021)	Randomized clinical trial	Test 1: root surface debridement (RSD) Test 2: ICG-aPDT/RSD in three groups: 1. non-diabetic, 2. well-controlled type 2 DM, 3. poorly-controlled type 2 DM	Chronic periodontitis	Indocyanine green 0.5 mg/ml	Diode laser: 810 nm, 200 mW, 4 J, papilla for 30 s followed by the insertion inside the periodontal pocket depth for 10 s from both buccal and lingual Single session	*Porphyromonas gingivalis, Tannerella forsythia*	3 and 6 months	Reduction for BOP PD, improvement CAL was noted for ICG-aPDT group. In all the three groups at 6 months reduction in both bacteria for ICG-aPDT group.
Alqerban (2020)	Randomized clinical trial	Test 1: ultrasonic scaling + aPDT Test 2: ultrasonic scaling + PBM Test 3: ultrasonic scaling	Gingivitis	Methylene blue, 0.0005%	Diode laser: 670 nm, 150 mW, 22 J/cm^2^, 60 s Single session	*Treponema denticola, Fusobacterium nucleatum* spp.	Baseline, 1 and 2 months	aPDT and PBM showed similar improvement in gingival inflammatory and microbiological parameters compared to ultrasonic scaling.
AlSarhan et al. (2021)	Randomized clinical trial	Test 1: SRP + aPDT Test 2: SRP	Chronic periodontitis	Indocyanine green, 0.1 mg/ml	Diode laser: 808 nm, 300 mW, 1414.7 J/cm^2^ Three sessions	23 bacterial species	Baseline, 1 and 3 months	A significant reduction in periodontal clinical parameters and microbial burden was seen in the aPDT group.
Alvarenga et al. (2019)	Randomized clinical trial	Test 1: 1 min aPDT + surfactant Test 2: 3 min aPDT + surfactant Test 3: 5 min aPDT + surfactant Test 4: 1 min aPDT Test 5: 3 min aPDT Test 6: 5 min aPDT	Chronic periodontitis	Methylene blue, 1 μM	Red laser: 660 nm, 100 mW, 75 J/cm^2^, 1, 3, 5 min Single session	NR	Immediately after irradiation	Methylene blue in the surfactant vehicle produced microbial reduction in the group irradiated for 5 min. Spectroscopy showed that surfactant vehicle decreased the dimer peak signal at 610 nm.
Arsic et al. (2022)	Randomized clinical trial	Test: SRP + aPDT Control: SRP	Chronic periodontitis	Phenothiazine chloride	Laser light: 660 nm, 100 mW, NR, 10 s Single session	*Aggregatibacter actinomycetemcomitans, Porphyromonas gingivalis,* and *Treponema denticola*	7 days	aPDT + SRP led to a more significant improvement in both clinical and microbiological assessments compared to SRP alone.
Arweiler et al. (2014)	Randomized clinical trial	Test: SRP + aPDT Control: SRP + 375 mg AMX and 250 mg MTZ	Aggressive periodontitis	Phenothiazine chloride	Diode laser: 660 nm, NR, NR, 60 s Two sessions	NR	3 months	Significant decreases in PPD, BOP, and CAL were found in two groups compared to baseline. Antibiotics significantly reduced PPD and CAL compared to aPDT.
Baeshen et al. (2020)	Randomized clinical trial	Test 1: scaling + aPDT Test 2: scaling	Gingivitis	Methylene blue, 0.005%	Diode laser: 670 nm, 150 mW, 22 J/cm^2^, 10 s Single session	*Porphyromonas gingivalis, Tannerella forsythia*	Baseline, 1 and 4 weeks	aPDT had a positive effect in reducing the microbial load in established gingivitis in adolescent patients undergoing fixed orthodontic treatment.
Bechara Andere et al. (2018)	Randomized clinical trial	Test 1: UPD + CLM Test 2: UPD + aPDT Test 3: UPD + CLM + aPDT Control: UPD	Aggressive periodontitis	Methylene blue, 10 mg/ml	Diode laser: 660 nm, 60 mW, NR, 60 s Single session	NR	3 and 6 months	At 3 M, all test groups exhibited reduced PPD relative to the control. At 6 M, the reduction in PPD was greater in Test groups 1 and 3. Test 3 group presented a significant gain in CAL relative to Test 2 and control.
Betsy et al. (2014)	Randomized clinical trial	Test: SRP + aPDT Control: SRP	Chronic periodontitis	Methylene blue, 10 mg/ml	Diode laser: 655 nm, 1,000 mW, 60 mW/cm^2^, 60 s Single session	NR	2 weeks, 1, 3 and 6 months	aPDT reduced PPD and CAL at 3 and 6 M, reduced PI at 2 W, and improved GI and GB at 2 W, 1 M, and 3 M compared to control.
Cadore et al. (2019)	Randomized clinical trial	Test: aPDT + ST Control: ST only	Chronic periodontitis	Phenothiazine chloride, 10 mg/ml	Diode laser: 660 nm, NR, 60 mW/cm^2^, 60 s Four sessions	40 subgingival microbial species	2 and 5 months	aPDT resulted in a significant reduction in PPD at 5 M compared to control. CAL gain was significantly higher in the test group at 2 and 5 M. Changes in the subgingival microbiota were similar between the groups, but aPDT revealed a larger number of bacteria associated with periodontal disease at 5 M compared to control.
Carvalho et al. (2015)	Randomized clinical trial	Test 1: aPDT Test 2: Irrigation	Chronic periodontitis	methylene blue, 0.01%	Diode laser: 660 nm, 40 mW, 90 J/cm^2^, 90 s Four sessions	*A. actinomycetemcomitans, P. gingivalis, Treponema denticola, Tannerella forsythia*	Baseline, 3, 6, 9, and 12 months	There was no significant difference between test and control groups.
Castro Dos Santos et al. (2016)	Randomized clinical trial	Test: SRP + aPDT Control: SRP	Chronic periodontitis	Methylene blue, 0.005%	Diode laser: 660 nm, 60 mW, 129 J/cm^2^, 60 s Single session	NR	1, 3, and 6 months	No statistically significant differences were observed between the two groups regarding any of the evaluated clinical parameters.
Chitsazi et al. (2014)	Randomized clinical trial	Test: SRP + aPDT Control: SRP	Aggressive periodontitis	Toluidine blue, NR	Diode laser: 670–690 nm, 75 mW, NR, 120 s Single session	*A. actinomycetemcomitans*	3 months	Both groups significantly reduced the presence of A.a, PPD, CAL, BOP, PI, and GI compared to baseline.
Cláudio et al. (2021)	Randomized clinical trial	Test: SRP + aPDT Control: SRP	Chronic periodontitis	Methylene blue, 10 mg/ml	Diode laser: 660 nm, 100 mW, 157 J/cm^2^, 50 s Single session	*Porphyromonas gingivalis and Prevotella intermedia*	3 and 6 months	Both groups showed reduction in PPD. SRP + aPDT also had a reduction in the number of pockets with PD ≥5 mm and BOP, at 3- and 6-months follow-up.
Cosgarea et al. (2022)	Randomized clinical trial	Test 1: SI + aPDT Test 2: SI + LDD Control: SI	Chronic periodontitis	HELBO Blue	Laser light: 660 nm, 100 mW, NR, 10 s Two sessions	*A. actinomycetemcomitans, P. gingivalis, T. forsythia, T. denticola, Parvimonas micra, F. nucleatum, Camphilobacter, and Filifactor allocis*	12 months	All of the treatments had statistically significant improvements in clinical parameters without significant differences between groups.
Cosgarea et al. (2021)	Randomized clinical trial	Test 1: SI + aPDT Test 2: SI + LDD Control: SI	Chronic periodontitis	HELBO Blue	Laser light: 660 nm, 100 mW, NR, 10 s 2 sessions	*Aggregatibacter actinomycetemcomitans, Porphyromonas gingivalis, Tannerella forsythia, Prevotella intermedia, Treponema denticola, Fusobacterium nucleatum, Campylobacter rectus, and Filifactor allocis*	3 and 6 months	All of the treatments had statistically significant improvements in clinical parameters without significant differences between groups.
de Melo Soares et al. (2019)	Randomized clinical trial	Test 1: SRP + aPDT Test 2: SRP	Chronic periodontitis	Phenothiazine chloride, 10 mg/ml	Diode soft laser: 660 nm, 70 mW, 2.79 J/cm^2^, 10 s Four sessions	Counts of 40 bacterial species were performed in each sample, using the checkerboard DNA–DNA hybridization technique	Baseline, 14, 30, and 90 days	No significant difference was seen in terms of clinical parameters between study groups.
Grzech-Leśniak et al. (2019)	Randomized clinical trial	Test 1: SRP + aPDT Test 2: SRP	Chronic periodontitis	Toluidine Blue 0.1%	Diode laser: 635 nm, 200 mW, 117.64 J/mm^2^, 60 s Three sessions	8 bacterial species	Baseline, 3 and 6 months	The aPDT group, substantially reduced the inflammation, BOP, and burden of microorganisms compared to the control group.
Hokari et al. (2018)	Randomized clinical trial	Test: UPD + aPDT Control: UPD + minocycline gel	Chronic periodontitis	Methylene blue, 0.01%	Diode laser: 670 nm, 140 mW, NR, 60 s Two sessions	*Porphyromonas gingivalis (P.g)* and *Tannerella forsythia (T.f)*	1 and 4 weeks	Significant decreases in PPD, BOP, and CAL were found in two groups compared to baseline. Antibiotics significantly reduced CAL, P.g, and T.f at 1 W.
Hill et al. (2019)	Randomized clinical trial	Test: SRP + aPDT Control: SRP	Chronic periodontitis	Indocyanine green, 0.1 mg/ml	Diode laser: 808 nm, 100 mW, NR, 20 s Single session	*Aggregatibacter actinomycetemcomitans, Porphyromonas gingivalis (P.g), Prevotella intermedia (P.i), Tannerella forsythia,* and *Treponema denticola (T.d)*	2 weeks, 3 and 6 months	BOP, RAL, and PPD decreased significantly in both groups at 3 M. No significant difference was observed between groups. aPDT significantly decreased the SFFR at 2 W. At 6 M, significant effects for P.g. in both groups were detected in relation to the baseline. aPDT significantly reduced P.i and T.d compared to the control group.
Husejnagic et al. (2019)	Randomized clinical trial	Test 1: SRP + aPDT Test 2: SRP	Chronic periodontitis	Tolonium chloride, 12.7 μg/ml	LED: 635 nm, 750 mW, 14 J/cm^2^, 60 s Two sessions	Eleven periopathogenic bacteria were investigated with a polymerase chain reaction (PCR) DNA probe test kit	Baseline, 12 weeks	No significant difference was seen in terms of clinical parameters between study groups.
Joshi et al. (2020)	Randomized clinical trial	Test: SRP + aPDT Control: SRP	Chronic periodontitis	Indocyanine green, 1 mg/ml	Diode laser: 810 nm, 200 mW, NR, 30 s Single session	NR	3 months	aPDT resulted in significant improvement in PPD and CAL compared to control. A significant reduction in PI and SBI was observed in both groups.
Katsikanis et al. (2020)	Randomized clinical trial	Test: SRP + aPDT Control: SRP	Chronic periodontitis	Methylene blue, 1%	Diode laser: 670 nm, 350 mW, 445 mW/cm^2^, 60 s Three sessions	NR	3 and 6 months	BOP, CAL, PPD, and PI were significantly improved in all groups at 3 months and 6 months compared with baseline. There was no statistically significant difference between groups.
Martins et al. (2017)	Randomized clinical trial	Test: aPDT + ST Control: ST only	Chronic periodontitis	Phenothiazine chloride solution, 10 mg/ml	Red laser: NR, 70 mW, 28 mW/cm^2^, 10 s Single session	40 bacterial species	2, 3, and 5 months	aPDT presented a significantly higher decrease in PPD than the Control Group at 3 M. Test Group also demonstrated significantly fewer periodontal pathogens of red complex (*Treponema denticola*) at 5 M.
Mocanu et al. (2021)	Randomized clinical trial	Test 1: SRP + CHX Test 2: SRP + aPDT Control: SRP	Chronic periodontitis	Phenothiazine chloride	Laser light: 660 nm, 100 mW, 10 s Three sessions	*Aggregatibacter actinomycetemcomitans, Porphyromonas gingivalis, Tannerella forsythia,* and *Treponema denticola*	1 and 6 months	aPDT significantly reduced plaque index, bleeding index, probing depth and attachment loss after 6 months.
Mongardini et al. (2014)	Randomized clinical trial	Test 1: SRP + aPDT Test 2: SRP	Chronic periodontitis	Toluidine blue O, 0.1 mg/ml	Diode laser: 628 nm, 1,000 mW, 20 J/cm^2^, 10s Single session	*Aggregatibacter actinomycetemcomitan, Porphyromonas gingivalis, Treponema denticola, Tannerella forsythia, Fusobacterium nucleatum* spp., and *Prevotella intermedia*	Baseline, 1 week	Greater reductions in microorganisms’ burden were observed in the test group.
Monzavi et al. (2016)	Randomized clinical trial	Test: SRP + aPDT Control: SRP	Chronic periodontitis	Indocyanine green, 1 mg/ml	Diode laser: 810 nm, 200 mW, NR, 10 s Four sessions	NR	1 and 3 months	aPDT showed significant improvements in BOP, PPD, and FMBS. Regarding PI, FMPS, and CAL, no significant differences were observed between both groups.
Moreira et al. (2015)	Randomized clinical trial	Test: SRP + aPDT Control: SRP	Aggressive periodontitis	Phenothiazine chloride, 10 mg/ml	Diode laser: 670 nm, 75 mW, 250 mW/cm^2^, 60 s Four sessions	Periodontal pathogens such as *A. actinomycetemcomitans* and species of orange and red complexes	3 months	aPDT significantly decreased PPD, CAL, and periodontal pathogens of red and orange complexes compared to control.
Müller Campanile et al. (2015)	Randomized clinical trial	Test 1: ultrasonic debridement +2 aPDT irradiation Test 2: ultrasonic debridement +1 aPDT irradiation Test 3: ultrasonic debridement	Periodontitis (Patients Under maintenance phase)	Methylene blue, NR	Diode laser: 670 nm, 280 mW, NR, 60 s Single or double sessions	*Porphyromonas gingivalis, Aggregatibacter actinomycetemcomitans, Tannerella forsythia, Treponema denticola, Prevotella intermedia,* and *Parvimonas micra*	Baseline, 3 and 6 months	A single or double episodes of PDT had some additional benefit over ultrasonic instrumentation alone.
Niazi et al. (2020)	Randomized clinical trial	Test: SRP + aPDT Control: SRP + SP gel	Chronic periodontitis	Indocyanine green	Diode laser: 810 nm, 100 mW, NR, 60 s Single session	NR	3, 6 months	Only aPDT significantly decreased CAL in moderate pockets compared to baseline. Between-group comparisons were non-significant.
Novaes Jr. et al. (2012)	Randomized clinical trial	Test: aPDT Control: SRP	Aggressive periodontitis	Phenothiazine chloride, NR	Diode laser: 660 nm, NR, 60 mW/cm^2^, 10 s Single session	*A. actinomycetemcomitans, T. forsythia,* and *P. gingivalis*	3 months	aPDT reduced the presence of *A.a* significantly compared to SRP and baseline.
Petelin et al. (2015)	Randomized clinical trial	Test 1: hand SRP Test 2: ultrasonic SRP Test 3: ultrasonic SRP + aPDT	Chronic periodontitis	Phenothiazine chloride, NR	Diode laser: 660 nm, 60 mW, 60 s Three sessions	*Aggregatibacter actinomycetemcomitans, P. gingivalis, Prevotella intermedia, Tannerella, forsythia,* and *Treponema denticola*	Baseline, 3, 6, 9, and 12 months	Adjunctive use of aPDT substantially reduced the BOP and microbial burden compared to SRP alone. There were no differences in terms of PPD and CAL between the groups. aPDT resulted in a greater reduction of periodontal pathogens compared to mechanical debridement alone.
Queiroz et al. (2015)	Randomized clinical trial	Test 1: SRP + aPDT Test 2: SRP	Chronic periodontitis	Phenothiazine chloride, 10 mg/ml	Diode laser: 660 nm, 60 mW, 2.79 J/cm^2^, 10 s Single session	NR	Baseline, 7, 30, and 90 days	No differences were observed between groups. The adjunctive effect of aPDT did not warrant improvements on clinical parameters in smokers.
Soundarajan and Rajasekar (2022)	Randomized clinical trial	Test 1: SRP + Er, Cr: YSGG laser Test 2: SRP + aPDT Control: SRP	Chronic periodontitis	Methylene blue, 1%	Diode laser: 660 nm, 70 mW, 16.72 J/cm^2^, 10 s Single session	NR	3 months	SRP + Er,Cr:YSGG laser showed more improved clinical outcomes compared with aPDT + SRP and control.
Sreedhar et al. (2015)	Randomized clinical trial	Test 1: SRP + PS Test 2: SRP + aPDT 1 session Test 3: SRP + aPDT 3 sessions Control: SRP	Chronic periodontitis	Curcumin, 10 mg/g	Blue halogen curing light, 470 nm, NR, NR, 50 min Single or triple sessions	*Aggregatibacter actinomycetemcomitans, Porphyromonas gingivalis, and Prevotella intermedia*	Baseline, 1 and 3 months	Curcumin + three sessions of aPDT showed a high reduction in SBI and PPD. SRP alone had a significant reduction in microbial parameters after 2 months and 3 months postoperatively.
Srikanth et al. (2015)	Randomized clinical trial	Test 1: SRP + aPDT Test 2: SRP + laser without PS Control: SRP	Chronic periodontitis	Indocyanine green, 5 mg/ml	Diode laser: 810 nm, 0.7 W, NR, 5 s Single session	*Prevotella intermedia*, *Veillonella parvula*, *Fusobacterium nucleatum*, *Porphyromonas gingivalis*, and *Aggregatibacter actinomycetemcomitans*	1 week, 3 and 6 months	aPDT showed a significant decrease in the percentage of viable bacteria at 1 W compared to the other groups. Comparing CAL and PPD revealed nonsignificant differences in aPDT sites at 6 M.
Sufaru et al. (2022)	Randomized clinical trial	Test: SRP + aPDT Control: SRP	Chronic periodontitis	Indocyanine green, 5 mg/ml	Diode laser: 810 nm, 0.2 W, NR, 60 s Four sessions	NR	6 months	BOP, PD and CAL were more significantly reduced in SRP + aPDT group than SRP alone. PI and HbA1c levels showed no statistically significant difference between groups.
Tabenski et al. (2017)	Randomized clinical trial	Test: SRP + aPDT Control: SRP + minocycline hydrochloride microspheres	Chronic periodontitis	Phenothiazine chloride, NR	Diode laser: 670 nm, 75 mW, NR, 10 s Two sessions	*A. actinomycetemcomitans, P. gingivalis, Tannerella forsythia (T.f.),* and *Treponema denticola (T.d.)*	6 weeks, 3, 6, and 12 months	Significant decreases in PPD, BOP, and CAL were found in two groups compared to baseline. Between-group comparisons were nonsignificant. More reduction of *P.g* DNA copies were found in aPDT group.
Theodoro et al. (2018)	Randomized clinical trial	Test: SRP + aPDT Control: SRP + 400 mg MTZ and 500 mg AMX	Chronic periodontitis	Methylene blue, 10 mg/ml	Diode laser: 660 nm, 100 mW, NR, 48 s Three sessions	*Porphyromonas gingivalis, Prevotella nigrescens,* and *Prevotella intermedia*	3 and 6 months	Significant decreases in PPD, BOP, CAL, *P. intermedia*, and *P. nigrescens* were found in two groups compared to baseline. Between-group comparisons were non-significant.
Theodoro et al. (2017)	Randomized clinical trial	Test: SRP + aPDT Control: SRP + 400 mg MTZ and 500 mg AMX	Chronic periodontitis	Methylene blue, 10 mg/ml	Diode laser: 660 nm, 100 mW, NR, 48 s Three sessions	NR	3 months	Significant decreases in PPD, BOP, and CAL were found in two groups compared to baseline. aPDT significantly reduced CAL in the moderate pocket for intergroup comparison.
Vohra et al. (2018)	Randomized clinical trial	Test: SRP + aPDT Control: SRP	Chronic periodontitis	Methylene blue, 0.005%	Diode laser: 670 nm, 150 mW, NR, NR Single session	NR	6 and 12 weeks	Significant reduction in PPD of 4-6 mm and ≥7 mm was observed for aPDT group as compared to the SRP group at both 6 and 12 W. There was a significant difference in TNF-α and IL-6 levels for aPDT group at 12 W.
Wadhwa et al. (2021)	Randomized clinical trial	Test: SRP + aPDT Control: SRP	Chronic periodontitis	Indocyanine green, 250 μg/ml	Diode laser: 810 nm, 500 mW, NR, 5 s Single session	NR	3 and 6 months	PI, GI, SBI, PPD, CAL, and the mean colony forming units scores showed no statistically significant difference between groups at baseline, but at 3 M and 6 M they were significantly lower in aPDT group compared to control.

NR, not reported; nm, nanometers; mW, milliwatts; s, seconds; SRP, scaling and root planning; UPD, ultrasonic periodontal debridement; PS, photosensitizer; CLM, clarithromycin; SP, salvadora persica; MTZ, metronidazole; AMX, amoxicillin; ST, surgical periodontal treatment; PI, plaque index; GI, gingival index; GB, gingival bleeding; SBI, sulcus bleeding index; PPD, pocket probing depth, CAL, clinical attachment loss; BOP, bleeding on probing; RAL, relative attachment loss; FMBS, full-mouth bleeding score.

Previously Vohra et al. reviewed the results of seven clinical studies to assess the effect of adjuvant aPDT in aggressive periodontitis therapy, now mostly classified as sever stages of periodontitis or periodontitis with a molar incisor pattern. Five studies confirmed the development of aPDT as an adjuvant to SRP to manage aggressive periodontitis, while two studies revealed that antibiotic administration in association with SRP had better outcomes than adjuvant aPDT to SRP. In these studies, Diode lasers with wavelengths between 660 and 690 nm for 60 to 120 s were used (Vohra et al., 2016). aPDT has shown positive outcomes regarding chronic periodontitis treatment as well. Akram et al. has systematically reviewed the evidence regarding aPDT and laser irradiation as adjuvants to open flap debridement (OFD) in chronic periodontitis treatment. Improvements in periodontal parameters were observed when aPDT was added to OFD (Akram et al., 2020).

The effect of aPDT is not merely limited to the treatment phase. Ramanauskaite et al. have explored the studies on the impact of aPDT on patients under supportive periodontal treatment (SPT) in a systematic review. Within the limitations of assessed studies, the data indicated the following outcomes: single and multiple adjunctive usages of aPDT subsequent to the subgingival debridement (SD) yielded a substantial reduction in BOP in comparison with SD alone; multiple applications of aPDT did not improve the outcomes compared to a single application (Ramanauskaite et al., 2021). In an RCT conducted by da Cruz Andrade et al., patients with severe chronic periodontitis treated by non-surgical periodontal therapy who underwent aPDT during the maintenance phase showed reduced inflammatory mediators. aPDT was conducted using 1 ml of the PS methylene blue 0.01% and 660 nm diode laser with an energy density of 90 J/cm^2^ (da Cruz Andrade et al., 2017). Thus, aPDT could be beneficial to inflammation control during the maintenance phase.

Based on the search results of the current study considerable number of RCT have been conducted on the clinical efficacy of different aPDT protocols compared to conventional non-surgical methods of treatment for periodontitis with the majority reporting improvements in clinical parameters (Table 1). Many of the conducted RCTs have also compared the changes in pathogenic bacterial levels and indicating the effectiveness of aPDT therapy in reducing bacterial loads with follow-ups of at least 3 months.

Photosensitizers used in the available studies have been Phenothiazines, Toluidine blue, Methylene blue, used with wave lengths in the red range 660–690 for activation and Indocyanine green with a wavelength in the near infrared rang 808-810 nm. Interestingly in some studies although the adjunctive aPDT was shown to be effective compared to baseline, however, no significant difference could be found compared to conventional treatment methods (Monzavi et al., 2016; Tabenski et al., 2017; Theodoro et al., 2018). Some studies have compared aPDT with adjunctive antibiotics and reporting superior results for conventional antibiotics (Arweiler et al., 2014; Hokari et al., 2018). There are also studies that have reported aPDT to be more effective in improving clinical attachment levels in moderate depth pockets compared to the groups receiving adjunctive antibiotic or antimicrobial treatments (Theodoro et al., 2018; Niazi et al., 2020). Overall, it seems that most studies report superior results of adding aPDT to conventional periodontal debridement.

Some studies have evaluated adjunctive aPDT in patients with systemic conditions such as diabetes or smokers and patients undergoing orthodontic treatments and reporting additional benefits for such adjunctive treatments in controlling the periodontal status in these patients (Queiroz et al., 2015; Alshahrani et al., 2020; Al-Momani, 2021). Interestingly, Niazi et al. looked into the clinical efficacy of aPDT in treating necrotizing ulcerative periodontitis (NUP) in HIV seropositive patients. They discovered that aPDT administration (0.005% methylene blue as PS and 670 nm diode laser with an energy density of 22 J/cm^2^) added to SRP effectively improved clinical periodontal parameters and reduced bacterial levels among HIV-positive patients affected by NUP. Yet, the improvements were not more significant than those in HIV seronegative patients (Niazi et al., 2020). Further studies conducted in such patient groups could add to the evidence.

Irradiation parameters varied between studies and no specific setting could be recommended. Most studies use low output powers of less than 500 mW that is considered suitable for PDT. The trials presented follow-up reviews of at least 3 months in all studies and a few with long term follow ups of up to 12 months. The available evidence indicates that multiple sessions of aPDT improve clinical, immunological, and microbiological parameters more effectively than a single session (Ozog et al., 2016; Joseph et al., 2017).

Light sources such as diode lasers and LEDs have been commonly used in the periodontal field (Cieplik et al., 2014). However, these light sources are associated with drawbacks such as tissue overheating when used with incorrect power densities and a restricted wavelength spectrum (Nagata et al., 2012). It is worth mentioning that the ideal light source for aPDT should be inexpensive, easy to handle, and capable of producing a range of wavelengths without overheating the tissues (Konopka and Goslinski, 2007). The combination of visible light and water-filtered infrared-A (VIS + wIRA) has been investigated as a novel light source to fulfill the mentioned aims. Studies have shown that applying VIS + wIRA significantly reduced the amount of periodontal and intra-radicular bacteria (Karygianni et al., 2014; Al-Ahmad et al., 2016; Burchard et al., 2020). These light sources increase the oxygen partial pressure in the target tissue without overheating the external tissue layers (Jung and Grune, 2012; Künzli et al., 2013).

Overall, based on the available evidence, aPDT can be considered a safe adjunctive method to conventional mechanical therapy for treating periodontal diseases and eliminating periodontal pathogens (Chambrone et al., 2018). However, various light sources, irradiation settings, and protocols have been used in different studies and conducting future systematic reviews focusing on a certain wavelength and also on each of the several photosensitizer types may be more beneficial in investigating their therapeutic effects and identifying suitable and most effective irradiation parameters for each. Moreover, the study population and various confounding factors need to be considered to reach more heterogenous clinical studies.

One of the limitations of aPDT in treating periodontitis is the lack of oxygen in deep periodontal pockets. Since oxygen is a vital requirement in photochemical reactions of aPDT, the efficacy of this method is questionable in deep periodontal pockets. It has been argued that photothermal therapy using PSs such as indocyanine green and other PS that relay on oxygen independent mechanisms of action may be more effective in these cases (Yang et al., 2019). Limitation of tissue penetration depth of visible light is another limitation that has been tried to be addressed by designing novel PS (Shi et al., 2019) Future developments in PSs may lead to superior clinical outcomes and evidence that better supports aPDT application in treating periodontal disease and even maintaining periodontal health.

## Antimicrobial photodynamic therapy in peri-implant diseases

Peri-implant disease is caused by the accumulation of bacterial biofilm, which induces an inflammatory process that influences the soft and hard tissue surrounding the implant fixture (Tenenbaum et al., 2017). It can be divided into two main categories: peri-implant mucositis and peri-implantitis (Klinge et al., 2018). The inflammation at the early stage only impacts the soft tissues surrounding the implant, causing symptoms like redness and bleeding. This stage is diagnosed as peri-implant mucositis. If the biofilm does not get removed, the inflammation proceeds to the hard tissue resulting in bone loss known as peri-implantitis. If left untreated, peri-implantitis could lead to implant loosening and failure (Berglundh et al., 2018).

Similar to periodontitis, the red complex bacteria including *P. gingivalis*, *Tannerella forsythia*, and *Treponema denticola* are the most common associated with severe peri-implantitis (Lasserre et al., 2018). Sites with peri-implant mucositis have a significantly higher proportion of *Prevotella* spp., *Porphyromonas* spp., *Treponema* spp., and *Alloprevotella* spp. than the healthy sites (Philip et al., 2022).

When infection is limited to soft tissues, mechanical debridement of the implant’s supracrestal surface and surrounding tissues assisted by aPDT has shown higher efficacy in controlling the biofilm’s spread compared to mechanical debridement alone. Consequently, this method could be beneficial in preventing the progress of biofilm to hard tissues and implant fixture surfaces. The detoxification process becomes more complicated as the biofilm proceeds into deeper tissues (Tavares et al., 2017). The design and topography of the implant fixtures make the debridement by mechanical tools rather difficult. Furthermore, mechanical debridement might damage the implant’s surface. Therefore, less invasive therapies such as aPDT can be beneficial in completing the biofilm removal in peri-implantitis cases (Fraga et al., 2018). However, the light source and the type of applied photosensitizer during aPDT should be cautiously chosen so as to minimize the absorbed light by titanium and thus, prevent a significant increase in the implant’s body temperature (Suarez et al., 2013).

Numerous *in-vitro* studies have been conducted to inspect the effectiveness of aPDT on microbial biofilm on implant surfaces and have reported obliteration of bacterial biofilms and substantial reduction in periodontal pathogens such as *P. gingivalis, Prevotella intermedia, and A. actinomycetemcomitans* (Dörtbudak et al., 2001; Sayar et al., 2019). significant reductions in counts of *P. gingivalis* and *Tannerella forsythia* at a 6-months follow-up after aPDT (Bassetti et al., 2014).

Several clinical studies have reported successful results following aPDT in managing peri-implant infections and eliminating pathogenic bacteria (Table 2).

**Table 2:** aPDT treatment in patients with peri-implant diseases.

Author, year	Study design	Treatment groups	Investigated pathology	Photosensitizer, concentration	Light type and parameters (wavelength, power, power density, irradiation time) and frequency of irradiation	Microorganisms	Follow-up periods	Outcomes
Al Rifaiy et al. (2018)	Randomized clinical trial	Test: MD + aPDT Control: MD alone	Peri-implant mucositis	Methylene blue, 0.005%	Diode laser: 670 nm, 150 mW, NR, 60 s Single session	NR	3 months	There was a significant improvement in PI and PPD at the 12-week follow-up with respect to the baseline visit in both groups. There was a significant reduction in PI and PPD for aPDT as compared to control at 3 M. There was no statistically significant difference for BOP between groups at follow-up.
Bassetti et al. (2014)	Randomized clinical trial	Test: MD + aPDT Control: MD + LDD	Peri-implantitis	Phenothiazine Chloride, NR	Diode laser: 660 nm, 100 mW, NR, 10 s Two sessions	*Porphyromonas gingivalis (P.g), Tannerella forsythia (T.f), Treponema denticola, Aggregatibacter actinomycetemcomitans, Prevotella intermedia, Campylobacter rectus, Fusobacterium nucleatum, Capnocytophaga gingivalis, Parvimonas micra, Eubacterium nodatum,* and *Eikenella corrodens*	3, 6, 9, and 12 months	PPD significantly decreased compared to baseline at aPDT-treated sites up to 9 months and up to 12 months at LDD-treated sites. Counts of P.g and T.f decreased significantly from baseline to 6 months in the aPDT and to 12 months in the LDD group, respectively. *CF* levels of IL-1b decreased significantly from baseline to 12 months in both groups. No significant differences were observed between groups after 12 months with respect to clinical, microbiological and host-derived parameters.
De Angelis et al. (2012)	Randomized clinical trial	Test: MD + aPDT Control: MD alone	Peri-impactites	Tolouidine blue O, 0.1 mg/ml	LED: 630 nm, NR, NR, 80 s Single session	NR	1 week, 1 and 4 months	PPD, BOP, and PI decreased in both groups without significant difference between them.
Javed et al. (2017)	Randomized clinical trial	Test: MC + aPDT Control: MC alone	Peri-implant mucositis	Phenothiazine Chloride, NR	Diode laser: 660 nm, 100 mW, NR, 10 s Single session	NR	3 months	PI, BOP, and PPD were comparable in both groups at baseline. At 3 M, there was a significant reduction in PI and PPD in test and control groups compared with their respective baselines. At 3 M, PI and PPD were significantly higher in the aPDT group compared to the control group. BOP was comparable in both groups at baseline and at 3 M.
Karimi et al. (2016)	Randomized clinical trial	Test: closed surface scaling + aPDT Control: closed surface scaling	Peri-impactites and peri-implant mucositis	Toluidine blue, 0.01%	LED: 630 nm, NR, 2000 mW/cm^2^, 120 s Single session	NR	1.5 and 3 months	There were significant differences in PPD, CAL, BOP, and GI at each time point between the two groups. There were no statistically significant changes with respect to any of the parameters in the control group. Complete resolution of BOP at 3 M was achieved in 100% of test implants. At 1.5 and 3 months, there were significant differences in the PPD and CAL gain in the test group.
Zeza et al. (2018)	Randomized clinical trial	Test: PAPR + aPDT	Peri-implant mucositis	Toluidine blue O, NR	LED: 630 nm, NR, NR, 10 s Single session	NR	2 and 6 weeks	Treatment with PAPR and aPDT resulted in a significant reduction in the BOP.

NR, not reported; nm: nanometers; mW, milliwatts; s, seconds; MC, mechanical curettage; MD, mechanical debridement; LDD, local drug delivery; PAPR, professionally administered plaque removal; SBI, sulcus bleeding index; CAL, clinical attachment loss; DIB, distance from implant to bone; PPD, pocket probing depth; BOP, bleeding on probing; PI, plaque index.

The effect of adjunctive aPDT in treating peri implant disease in patients with systemic risk factors such as smoking has also been a topic of clinical research. Al Rifaiy et al. showed that aPDT is more effective than mechanical debridement alone in treating peri-implant mucositis in e-cigarette (vaping) users (Al Rifaiy et al., 2018). Javed et al. also stated the superiority of aPDT and mechanical curettage (MC) in treating peri-implant mucositis in cigarette smokers compared with MC alone (Javed et al., 2017). According to Almohareb et al.’s study, aPDT adjuvant to mechanical debridement was as effective as conventional antimicrobial therapy in reducing severe peri-implant symptoms. aPDT was applied *via* a 670 nm diode laser and methylene blue as the required PS (Almohareb et al., 2020). There are also positive effects of aPDT in treating peri-implant diseases in patients with predisposing factors. Alqahtani et al. reported mechanical debridement with adjunct aPDT to effectively treat peri-implantitis in smokers. 0.005% of MB was applied into the pocket and later irradiated with a 660 nm diode laser at a power output of 150 mW and energy fluency of 0.0125 J/cm^2^ (Alqahtani et al., 2019). In addition to the therapeutic effects of aPDT on clinical outcomes and bacterial loads, some studies have investigated the preventive impact of aPDT on reducing inflammatory cytokines following implant insertion. Zhou et al. observed a significant reduction in cytokine levels (IL-1β, ΤΝF-α, IL-6, and ΙL-17) of the group treated with aPDT right after the completion of implants’ supra-structures (Zhou et al., 2019).

Immediate implant placement is a technique that reduces treatment time, facilitates the healing period, and improves esthetic results. Although, this method comes with the risk of infection spreading to the implant’s surrounding tissues in cases of active infection in extraction sockets (Quirynen et al., 2005). It is worth mentioning that aPDT has shown to be effective in reducing periapical infection in these cases and thus elevating the success rate of immediately inserted implants in infected sockets receiving this adjunctive treatment (Alghandour et al., 2018).

Several studies have investigated the effect of aPDT on peri-implantitis’ treatment outcomes. Romeo et al. showed that aPDT as a co-adjuvant in the treatment of peri-implantitis associated with mechanical (scaling) and surgical (grafts) treatments resulted in a better value in terms of PPD, BOP, and PI after 6 months compared with mechanical and surgical therapies alone. They used a 670 nm diode laser with an output of 75 mW/cm^2^ and 10 mg/ml methylene blue as the PS (Romeo et al., 2016). Two trials concluded that aPDT combined with mechanical debridement is as effective as local antibiotic delivery with mechanical debridement (Bassetti et al., 2014). However, some studies have reported that other disinfection methods showed superior results compared to aPDT. For example, Birang et al.’s study revealed the antibacterial effect of 2% chlorhexidine on biofilm of *A. actinomycetemcomitans* to sandblasted, large-grit, acid-etched (SLA) implant surfaces to be greater than aPDT (660 nm diode laser with an energy density of 5 J/cm^2^) and Er.YAG laser effects. Although, aPDT displayed higher antibacterial effects compared to disinfection with Er.YAG laser (Birang et al., 2019).

One of the advantages of using aPDT in peri-implant diseases discovered in experimental studies is that, unlike mechanical debridement, aPDT is less likely to damage the implant’s surface during the detoxification process. In a confirming study, Saffarpour et al. examined the microstructure of contaminated implants after aPDT (630 nm light-emitting diode with toluidine blue O as PS, 810 nm diode laser with indocyanine green as PS) by scanning electron microscopy and energy-dispersive x-ray spectroscopy, which depicted no alterations to the surface of treated implants (Saffarpour et al., 2018).

A network meta-analysis has been conducted on RCTs that compared aPDT and other treatments in individuals with peri-implantitis. These studies depicted a substantial reduction in the values of clinical attachment through aPDT combined with mechanical debridement in comparison with other treatments investigated. However, no statistically significant results were observed for BOP, PD, and plaque scores (Sivaramakrishnan and Sridharan, 2018). The impact of aPDT on peri-implant mucositis has also been investigated in a systematic review. The included studies revealed improved inflammation around dental implants. However, the definite conclusion was hindered by heterogeneity in laser parameters, control groups, and follow-up periods. Hence, further well-designed studies with standardized parameters are required (Albaker et al., 2018). Moreover, in a recent review of RCTs, aPDT decreases bacterial load associated with peri-implant diseases and may be considered an alternative to antibiotics (Rahman et al., 2022).

The available clinical studies have used Phenothiazine Chloride, Toluidine blue and Methylene blue activated with wavelengths of 630-660 nm. Most studies have used aPDT in only one session.

Based on the available RCT, there are some studies reporting positive effects on clinical outcomes with aPDT treatment and others only showing aPDT to be effective in improving clinical outcomes and reducing bacterial loads compared to baseline without any significant difference compared to the control groups receiving conventional treatment.

Overall, aPDT can be considered a promising treatment for peri-implant infections. Due to its local effects and non-invasive on implants. However, like most treatment procedures, cases of peri-implant diseases need to be carefully selected and administration parameters should be set appropriately. Further studies are still needed implementing similar protocols as much as possible to add the evidence that can guide clinicians.

## Applications of antimicrobial photodynamic therapy in endodontics

Various bacterial strains can be associated with endodontic infections, such as *Streptococcus* spp.*, Peptostreptococcus* spp.*, Lactobacilli* spp.*, Propionibacterium* spp.*, Actinomyces* spp.*, Eubacterium* spp.*, Veillonella parvula, Bacteroides* spp., *Fusobacterium* spp., and *Enterococcus faecalis* (Siqueira and Rôças, 2014). During primary apical periodontitis, intra-radicular areas of the teeth are mainly occupied by obligate anaerobic species. On the other hand, secondary apical periodontitis is characterized by the dominant presence of both anaerobes and facultatives (Siqueira Jr. and Rôças, 2022). Microbial contamination of the root canals can proceed beyond the pulp tissue space to the dentinal tubules. Thus, mechanical decontamination and chemical irrigation methods cannot eradicate bacterial contamination, especially considering limitations such as accessory canals, anastomoses, and the root canal complex anatomy. Using PS molecules with aPDT has shown to be a promising alternative for endodontic disinfection (Abdelkarim-Elafifi et al., 2021). Commonly used PSs in treating endodontic infections are hematoporphyrin derivatives, toluidine blue O, methylene blue, cyanine, phthalocyanine, and phototherapeutic agents (Meisel and Kocher, 2005; Sigusch et al., 2005; Pinheiro et al., 2009). Among them, phenothiazine salts like toluidine blue O and methylene blue are more popular since they can stain both Gram-negative and Gram-positive bacteria responsible for most endodontic infections (Gajdács et al., 2017). The most common wavelength that activated these PSs ranged between 630 and 700 nm (Giusti et al., 2008). We should mention that a wide range of power settings (40 to 100 mW) and exposure times (60 to 240 s) have been used in the literature (Foschi et al., 2007; Garcez et al., 2008; Garcez and Hamblin, 2017). Thus, the differences in culture protocols and aPDT parameters deter comparison between studies. The available clinical studies in this field have mostly used Phenothiazinium Chloride, Methylene blue and Toluidine blue as a PS activated with LED or laser light sources in the red range wavelength. Irradiation parameters are different in each study.

It is worth mentioning that the application of aPDT has its drawbacks. The structure of dentinal tubules with 1–2 μm lumen and 2–3 mm length causes serious challenges for all disinfection methods. aPDT is not an exception as the light propagation and PS penetration inside dentinal tubules are restricted. Moreover, reports have shown that bacteria can migrate into dentinal tubules up to a depth of 1,000 μm, where oxygen as a vital component of aPDT is absent (Balhaddad et al., 2020; Rosen et al., 2020). To overcome these issues, some studies used hydrogen peroxide solution that provided pre-treatment of the biofilm, resulting in better PS penetration and increased available oxygen in the environment (Garcez et al., 2011; Garcez and Hamblin, 2017). Recently, nanoparticles (1–100 nm) have been introduced as emergent PS carriers that provide many advantages in favor of the antimicrobial efficacy of aPDT over conventional PSs. These nanoparticles can be diversely designed and conveniently penetrate into dentinal tubules (Misba et al., 2016; Alfirdous et al., 2021).

Studies have shown that aPDT makes significant bacterial elimination possible, even in cases with antibiotic-resistant species. Several *in-vitro* studies have confirmed the positive effect of adding aPDT to conventional irrigation methods (sodium hypochlorite or chlorhexidine) on *Enterococcus faecalis* reduction (Ghorbanzadeh et al., 2018; Asnaashari et al., 2020). There is also promising evidence of the lethal effect of aPDT on *Enterococcus faecalis* in both primary endodontic treatments and re-treatments (Tennert et al., 2014). Vendramini et al. have systematically reviewed *in-vitro* studies about the antimicrobial effect of aPDT on intracanal biofilm and concluded that aPDT reduced bacterial amounts in most studies, particularly when assisted by the conventional endodontic techniques to treat refractory infection (Vendramini et al., 2020).

Clinical studies on this topic recommend that aPDT can be a promising treatment modality for reducing bacterial complications (Table 3). In a recent retrospective clinical study by Conejero et al., 100 teeth were treated with conventional chemo-mechanical disinfection (CMD) on either a primary or re-treatment basis, and 114 teeth received CMD + aPDT. aPDT was applied using 0.1 mg/ml toluidine blue PS and 630 nm LED at 2000 mW/cm^2^. The CMD + aPDT group showed a shorter periapical healing time (15 ± 9.33 months) and higher success rate (97.2%) compared to CMD alone healing time (20.35 ± 22.1 months) and success rate (94.7%; Conejero et al., 2021).

**Table 3:** aPDT treatment in patients with endodontic infection.

Author, year	Study design	Treatment groups	Investigated pathology	Photosensitizer, concentration	Light source parameters (wavelength, power, power density, irradiation time) and frequency of irradiation	Microorganisms	Follow-up periods	Outcomes
Ahangari et al. (2017)	Randomized clinical trial	Test 1: CMD + aPDT Test 2: CMD + Ca (OH)_2_ therapy	Persistent endodontic infection	Methylene blue, 0.05 mg/ml	Diode laser: 810 nm, 200 mW, NR, 10 s Single session	*Enterococcus faecalis* and *Candida albicans*	2 weeks	aPDT presented similar CFU/ml reduction compared with Ca (OH)_2_ therapy.
Alves-Silva et al. (2022)	Randomized clinical trial	Test: CT + aPDT Control: CT	Primary endodontic infection	Methylene blue, 0.005%	Diode laser: 660 nm, 100 mW, 320 J/cm^2^, 90 s Single session	NR	8, 12, 24, 48, and 72 h, 1 week	There was a statistically significant difference (*p* < 0.05) in the periods of 8, 12, 24, 48 and 72 h between the control group and the aPDT group. After 1 week, there was no statistically significant difference.
Asnaashari et al. (2017)	Randomized clinical trial	Test 1: CT + aPDT Test 2: CT + Ca (OH)_2_ therapy	Persistent endodontic infection	Tolouidine blue O, 0.1 mg/ml	LED: 635 nm, NR, 2–4 mW/cm^2^, 60 s Single session	*Enterococcus faecalis*	2 weeks	The number of CFU/ml was lower in aPDT compared with Ca (OH)_2_ therapy
Coelho et al. (2019)	Randomized clinical trial	Test: CT + aPDT Control: CT	Primary endodontic infection	Methylene blue, 1.56 μM/ml	Diode laser: 660 nm, 100 mW, 600 J/cm^2^, 180 s Single session	NR	24 and 72 h, 1 week	Postoperative pain was significantly decreased after aPDT at 24 and 72 h intervals.
da Silva et al. (2018)	Randomized clinical trial	Test: CMD + aPDT Control: CMD	Primary endodontic infection	Methylene blue, 0.1 mg/ml	Diode laser: 660 nm, 100 mW, NR, 40 s Single session	*Enterococcus faecalis*, *Candida* genus and Bacteria domain	1 week	aPDT resulted in a significant reduction in the incidence of *E. faecalis* before root canal obturation at the second session in teeth with primary endodontic infections.
Di Taranto et al. (2022)	Randomized clinical trial	Test 1: CT + high-power laser Test 2: CT + aPDT	Primary endodontic infection	Toluidine blue O, 155 μg ml	Diode laser, 660 nm, 100 mW Single session	*Enterococcus* sp.*, Candida* sp.*, Lactobacillus* sp. *and Phorphyromonas* sp.	1 week	The difference between CFUs before and after aPDT protocol was significant. Further statistically significant CFU reduction was seen after the second laser treatment in the aPDT group.
Guimaraes et al. (2021)	Randomized clinical trial	Test: CT + aPDT + LLLT Control: CT	Primary endodontic infection	Methylene blue, 0.01%	Diode laser: 660 nm, 100 mW, 300 J/cm^2^, 90 s Single session	NR	2, 3, and 7 days	There were no significant differences in post-operative pain, tenderness, oedema and the use of analgesics between groups at any observation period.
Jurič et al. (2014)	Randomized clinical trial	Test 1: endodontic treatment with 2.5% NaOCl and 17% EDTA Test 2: aPDT Test 3: CT	Persistent endodontic infection	Phenothiazinium Chloride, 10 mg/ml	Diode laser: 660 nm, 100 mW, NR, 60 s Single session	*Enterococcus faecalis, Peptostreptococcus, Actinomyces naeslundii, Actinomyces odontolyticus, Porphyromonas, Veillonella parvula,* and *Pseudomonas aeruginosa*	2 weeks	The combination of endodontic treatment and aPDT was statistically more effective than endodontic treatment alone.
de Miranda and Colombo (2018)	Randomized clinical trial	Test: CMD + aPDT Control: CMD	Primary endodontic infections	Methylene blue, 25 μg/ml	Diode laser: 660 nm, 100 mW, NR, 300 s Single session	*Candida albicans*, *Dialister pneumosintes*, *Prevotella nigrescens*, *Prevotella tannerae*, *Parvimonas micra*, *Peptostreptococcus anaerobius*, *Propionibacterium acnes,* and others	3 and 6 months	aPDT presented a similar CFU/ml reduction compared with control. Significant decreases in PAI scores were observed in both groups over time, although at 6 M, the PDT group presented a significantly better healing score than the control. *C. Albicans* and *D. Pneumosintes* were still detected in high frequency in both groups at 3 M.
Moreira et al. (2021)	Randomized clinical trial	Test: CT + intracanal medication + aPDT Control: CT + intracanal medication	Primary endodontic infection	Methylene blue, 0.005%	Red laser: 660 nm, 90 s Two sessions	*Enterococcus faecalis* and *Actinomyces israelii*	2 months	aPDT did not show better results, in comparison with conventional treatment.
Okamoto et al. (2020)	Randomized clinical trial	Test: CT + aPDT Control: CT	Primary endodontic infection	Methylene blue, 0.005%	660 nm, 100 mW, 4 J/cm^2^, 40 s Single session	Total number of viable bacteria	1 and 3 months	The difference between the control and test groups was not significant.
Pourhajibagher and Bahador (2018)	Randomized clinical trial	Test: aPDT Control: CT	Primary endodontic infection	Tolouidine blue O, 0.1 mg/ml	Diode laser: 635 nm, 220 mW, NR, 60 s Single session	*V. parvula, P. gingivalis, Propionibacterium acnes, Lactobacillus acidophilus, C. rectus, S. exigua, A. actinomycetemcomitans, Pseudomonas aeruginosa, Actinomyces naeslundii, L. rhamnosus, L. casei, Candida albicans, P. aeruginosa, Enterococcus faecalis, Streptococcus sanguinis, A. naeslundii, S. salivarius, S. mitis, C. rectus, K. pneumoniase, S. epidermidis,* and *S. mutans*	NR	There was a significant decrease in the microbial diversity and count of the infected root canal after aPDT.

NR, not reported; nm: nanometers; mW, milliwatts; s, seconds; CT, conventional endodontic therapy; CMD, chemo-mechanical debridement; SSL, saline solution; LLLT, low-level laser therapy.

Few RCTs on aPDT concerning endodontic treatments have been done. De Miranda et al. reached boosted healing and lower periapical index (PAI) points at the 6-month follow-up after treating necrotic teeth with aPDT. They injected 0.5 ml of 25 μg/ml MB into the canals, followed by a 660 nm diode laser irradiation at 100 mW (de Miranda and Colombo, 2018). Juric et al. investigated the value of aPDT (PS: phenothiazinium chloride; 660 nm diode laser at 100 mW power) in patients with root-filled teeth and infected root canal systems accompanied by chronic apical periodontitis. They found that conventional endodontic therapy followed by aPDT reached a significant additional reduction of intracanal microbial load (Jurič et al., 2014).

Systematic reviews on this topic have confirmed its therapeutic advantages in endodontics. Pourhajibagher et al. conducted a systematic review and meta-analysis to evaluate the effect of combined aPDT and conventional chemo-mechanical debridement of infected root canal systems in patients with endodontic infections. They found a decrease in microbial load with the adjunctive application of aPDT in all studies; nevertheless, more RCTs with robust designs to focus on coordinating applicated aPDT parameters were suggested (Pourhajibagher and Bahador, 2019).

An interesting finding of the RCTs is the benefits of aPDT in a single session. Rabello et al., who investigated the antimicrobial effect of aPDT in a single visit versus two-visit cases with calcium hydroxide intracanal medication between appointments, reported a significant bacterial reduction in single-visit patients treated with aPDT with no further improvements in the two-visit method. They applied 0.1 mg/ml of MB to root canals, which were subsequently irradiated with a 660 nm diode laser at a fluency of 129 J/cm^2^ (Rabello et al., 2017). Another RCT by Asnaashari et al. concerning endodontic re-treatment cases revealed even a greater microbiological elimination after using aPDT in a single session compared to a calcium hydroxide covering in two sessions. After the 0.5 ml toluidine blue (0.1 mg/ml) application into canals, they were irradiated with a 630 nm LED at the fluency of 1.2–4.4 mJ/cm^2^ (Asnaashari et al., 2017). Hence, a single visit completed endodontic treatment enables instant coronal restoration, decreasing potential bacterial contamination from the oral microbial flora over the waiting interval between two sessions.

aPDT may be considered an antibacterial alternative to systemic and local antibiotics in endodontics due to the lack of bacterial resistance reported with aPDT so far (Abdelkarim-Elafifi et al., 2021).

## Anticaries and antiplaque applications of antimicrobial photodynamic therapy

There have been debates on the necessary amount of carious dentin that needs to be removed before restorative treatment in carious dentin in recent years. Maximum tissue preservation, particularly in managing deep carious lesions, is highly recommended to prevent potential pulp exposure. Disinfection of remaining affected dentin using minimally invasive approaches such as aPDT can improve the treatment prognosis by inactivating the cariogenic bacteria while preserving tooth structure (Rolim et al., 2012; Alves et al., 2019; Bargrizan et al., 2019). The rate of success in microbial inactivation depends on factors like light dosimetry, incubation time, and PS penetration in the targeted cells, which is determined by the charge, size, and solubility of the substance (Rolim et al., 2012; Pogue et al., 2016). Many studies have investigated the phenothiazinium dyes methylene blue and toluidine blue O because of their ability to generate a high singlet oxygen amount, strong absorption in the red-light spectrum (600–680 nm), and also reducing bacterial matrix polysaccharides (Pereira et al., 2011; Vahabi et al., 2011; Felgenträger et al., 2013; Manoil et al., 2014; Cusicanqui Méndez et al., 2018). Other investigated substances are curcumin, indocyanine green, rose bengal, fotoenticine, and some other PSs which have shown a positive antimicrobial effect on dentin caries (Soria-Lozano et al., 2015; Azizi et al., 2016; Cusicanqui Méndez et al., 2018; Alfirdous et al., 2021; Table 4).

**Table 4:** Anticaries aPDT treatment.

Author/year	Study design	Study groups	Photosensitizer type/concentration	Light type and irradiation parameters	Microorganism	Follow-up periods	Outcomes
Alsaif et al. (2021)	Randomized clinical trial	Control 1: no Erythrosine, no light Control 2: Erythrosine, no light Test 1: aPDT (continuous light) Test 2: aPDT (pulsed)	Erythrosine, 220 μM	Tungsten filament Lamp: 500–550 nm, 400 W, 22.7 mW/cm^2^, 15 min, or 5*30 s Single session	NR	2 weeks	Treatment groups had significantly higher reduction in their CFU compared to the control groups. No statistically significant difference was observed between the four treatment groups. Using either 2 min or 15 min incubation times after 15 min continuous irradiation showed Significant reductions in the CFU count.
Alves et al. (2019)	Randomized clinical trial	Test: aPDT Control: non-aPDT treatment	Methylene blue, 0.005%	Diode laser: 660 nm, 100 mW, 640 J/cm^2^, 180 s Single session	*Streptococcus mutans*	6 months	aPDT treatment following caries removal showed a significant reduction in the *S. mutans* CFU compared to the control group. aPDT had no considerable effects regarding retention, marginal adaptation and discoloration, secondary caries, and color of the restoration compared to the control group.
Ichinose-Tsuno et al. (2014)	Randomized clinical trial	Test: aPDT Control: without aPDT	Toluidine blue O, 1 mg/ml	Red LED: 600–700 nm, 20 s Six sessions	NR	4 days	Plaque deposition areas and the total number of bacteria in the dental plaque were considerably reduced in the aPDT group compared to the control group.
Lima et al. (2022)	Randomized clinical trial	Test 1: biofilm before aPDT Test 2: biofilm 1 min after aPDT Test 3: biofilm before aPDT Test 4: biofilm 5 min after aPDT	Methylene blue, 0.01%	Diode laser: 660 nm, 90 J/cm^2^, 1.1 W/cm^2^, 100 s Single session	NR	NR	Both treatment groups demonstrated a decrease in the number of bacteria. The most evident reduction was noticed in the group with a 5 min pre-irradiation time.
Longo et al. (2012)	Randomized clinical trial	Test: ART + aPDT Control: ART	Aluminum-chloride-phthalocyanine (AlClPc),	Red laser: 660 nm, 180 J/cm^2^, 250 mW/cm^2^, 180 s Single session	NR	NR	Cationic liposomes containing AlClPc as PS was able to efficiently reduce the bacterial count in an infected dentin and has enough safety for clinical use.
Melo et al. (2015)	Randomized clinical trial	Test: aPDT Control: non-aPDT treatment	Toluidine blue O, 100 μg/ml	LED: 630 nm, 150 mW, 94 J/cm^2^ Single session	*Streptococcus mutans, Lactobacillus* spp.	NR	Cariogenic microbial load in deep dentinal caries was significantly decreased after aPDT.
Steiner-Oliveira et al. (2015)	Randomized clinical trial	Test: aPDT with TBO Test 2: aPDT with MB Control: chlorhexidine	Toluidine blue O, 0.1 mg/ml Methylene blue, 0.01%	LED: 630 nm, 30 J/cm^2^, 60 s Single session Red laser: 660 nm, 320 J/cm^2^, 90 s Single session	*Streptococcus mutans, Streptococcus sobrinus, Lactobacillus casei, Fusobacterium nucleatum, Atopobium rimae*	6 and 12 months	There were no statistically significant differences among the three protocols.
Tahmassebi et al. (2015)	Randomized clinical trial	Group 1: Control + aPDT test (varied concentrations of PS) Group 2: Control + aPDT test (varied light dose)	Erythrosine, 22 and 220 μM	Tungsten filament Lamp: 535 nm, 400 W, 22.7 mW/cm^2^ Single session	NR	2 weeks	Group 1: Bacterial reduction was observed in an erythrosine dose-dependent manner. The total bacterial counts were significantly lower in the 220 μM erythrosine group compared with the 22 μM group. Group 2: Bacterial reduction was observed in an erythrosine light dose-dependent manner. 15 min continuous irradiation and cut-off light irradiation of 5 * 1 min were the most effective regimens for reducing bacteria with 220 μM erythrosine.

NR, not reported; nm, nanometers; μM, micrometer; mW, milliwatts; s, seconds; PS, photosensitizer: ART, atraumatic restorative treatment.

In this review, eight randomized clinical studies were identified, evaluating anticaries effect of aPDT with varying clinical protocols, however, there are other clinical studies available that do not have a randomized design. Toluidine blue O, Methylene blue, Aluminum-chloride-phthalocyanine (AlClPc) and Erythrosine were used as PS in these studies.

Melo et al. performed a randomized, controlled, split-mouth clinical trial to investigate the efficacy of deep dentinal caries disinfection with aPDT. The number of viable *S. mutans* and *Lactobacillus* spp. were significantly reduced after being exposed to 100 μg/ml toluidine blue O for 5 min and 630 nm LED with a total energy density of 94 J/cm^2^ indicating the improvement in carious dentin disinfection following aPDT (Melo et al., 2015). A significant decrease was seen in the viability of *S. mutans*, *L. casei*, and *A. viscosus* in both planktonic and sessile forms in an *in-vitro* study by Darmani et al. using a GaAlAs laser at 670 nm and toluidine blue O as PS (Darmani et al., 2018).

Methylene blue was the subject of anticaries aPDT in some clinical studies (but not-randomized) conducted by Neves et al., Guglielmi et al., and Ornellas et al. evaluating the effect of aPDT on *S. mutans* and *Lactobacillus* ssp. resulting in controversial findings (Neves et al., 2016; Ornellas et al., 2018). Neves et al. conducted a case control study and microbiologically assessed the dentin samples obtained from the pulp wall of deciduous teeth before and after applying aPDT. They irradiated the cavity after taking the control sample and applying methylene blue using an InGaAlP laser with an energy density of 120 J/cm^2^. While they declared no statistically significant reduction in the count of viable carious microorganisms before and after application of PS, Guglielmi et al. reported that aPDT significantly decreased total viable bacteria of the permanent molar samples with an active deep carious lesion without pulpal involvement. Both mentioned studies used an InGaAlP laser with 660 nm wavelength and 5 min pre-irradiation time, but the energy density in the second study was higher and reported as 320 J/cm^2^.The energy density and the observed results in the clinical experiment of Ornellas et al. (InGaAIP, 660 nm, 300 J/cm^2^, 5 min) were more similar to that of Guglielmi et al.

A RCT by Steiner-Oliveria et al. compared aPDT with TB and MB using an LED 630 nm irradiation at 30 J/cm^2^ and a control treatment of Chlorhexidine on *S. mutans, S. sobrinus*, and *Lactobacilus casei*. The results revealed no statistically significant difference among study groups (Steiner-Oliveira et al., 2015). However, another RCT using TB as a PS and application of LED 600–700 nm irradiation in six sessions, reported considerable reduction in the total number of bacteria and plaque deposition in the aPDT treated group vs. controls without any aPDT (Ichinose-Tsuno et al., 2014).

Reducing the *S. mutans* count in the oral cavity by performing aPDT and before restoration placement may decrease the risk of caries reoccurrence and some studies have focused on these clinical applications of aPDT. Effect of aPDT on salivary *S. mutans* in 5- to 6-year-old children aPDT with severe early childhood caries using toluidine blue O and 633 nm diode laser was assessed in a case–control study by Bargrizan et al. Two sessions of aPDT were performed (20 mW, 6 J/cm^2^), and salivary samples were collected to be compared against the other groups. They concluded that the efficacy of toluidine blue O plus diode laser in reducing *S. mutans* count was higher than other groups that used only toluidine blue O, only laser, or none, and also the treatment was more durable after receiving two doses of aPDT (Bargrizan et al., 2019). aPDT has been demonstrated to prevent enamel demineralization even with the presence of a cariogenic diet, that indicates its effectiveness in caries prevention (Baptista et al., 2012).

Mendez et al. analyzed the influence of methylene blue on the viability of carious microorganisms and their lactic acid production. The highest reduction in the vitality of intact biofilms and the number of microorganisms was measured after using methylene blue with 75 J/cm^2^ fluence, and all treatment groups had significantly lower lactic acid production except when the methylene blue was used without illumination (Cusicanqui Méndez et al., 2018). In another study by Mendez et al., the combination of curcumin and LED laser irradiation significantly reduced the number of colony-forming units and vitality of intact biofilms, but it did not show any considerable drop in their acidogenicity feature (Cusicanqui Méndez et al., 2018).

Pereira et al. aimed to explore the effect of polyacrylic acid (PA) 11.5% containing 0.3% methylene blue as a PS to reduce the microbial load before restoration placement. Treatment with methylene blue and methylene blue + PA showed the most reduction in *S. mutans* growth, respectively, depicting that it can be used as a PS to diminish *S. mutans* carious dentin (Pereira et al., 2020). Pinheiro et al. also examined the use of a dental acid etchant containing 37% phosphoric acid and methylene blue (DAE) as a sensitizing agent in aPDT of dentinal caries.

The specimens were exposed to a 660 nm light irradiation with 4 J/cm^2^ energy density. The most significant relative reduction in the number of *S. mutans* was obtained in the PDT group and then the DAE group making it a potential PS to be used in future clinical studies (Pinheiro et al., 2019).

One of the investigation areas in restorative dentistry is the effect of aPDT on the bond stability of materials and the durability of the treatment. The less collagen content, loss of peritubular dentin matrix, and increased water in the carious affected dentine are some of the characteristics that compromise the success of the treatment by causing gap or leakage in the interface of the tooth and restoration and lowering the bond strength (Nakajima et al., 2011; Pinna et al., 2015), thus, it is strongly suggested to efficiently disinfect this layer in order to achieve suitable bond integrity and successful restoration (Perdigão et al., 2021). The conventional disinfection methods such as exploiting chlorhexidine gluconate, sodium hypochlorite, ethylene diamine tetra acetic acid, and hydrogen peroxide in the cavity have been shown to jeopardize the stability of the bond over time (Tulunoglu et al., 1998; Shafiei and Memarpour, 2012; Coelho et al., 2021).

Recently, aPDT has been investigated by many researchers and is considered an alternative non-invasive treatment for treating deep carious lesions for its promising results (Borgia et al., 2018; Alrahlah et al., 2020; Alshami et al., 2021; Hashemikamangar et al., 2022). In a study by Alrahlah et al. evaluating the effect of methylene blue, curcumin, indocyanine green, and H_2_O_2_ on shear bond strength (SBS) of composite resin restorations on carious dentin samples, the highest SBS value was detected in the samples disinfected by curcumin. Curcumin and indocyanine green demonstrated the potential to be used as PS since they can improve the SBS of restoration to carious tissues (Alrahlah et al., 2020). Keskin et al. compared the microtensile bond strength (μTBS) of giomer to carious dentin when disinfected with CHX, NaOCl, aPDT, or Er, Cr: YSGG laser before restoration placement (Keskin et al., 2021). Faria et al. clinically evaluated the performance of composite restorations after caries removal (SCR) associated with aPDT (Faria et al., 2022). The marginal adaptation of the restoration was significantly better in aPDT group compared to the control group after a 12-month follow-up.

Although all the disinfection protocols reduced the bond strength to caries-affected dentin, The aPDT and laser groups showed more μTBS values than the CHX and NaOCl (Keskin et al., 2021). Their results were in agreement with Vellappally et al.’s research indicating the effectiveness of aPDT in augmenting the bond strength. However, using aPDT as effective treatment has raised scientific debates as there have been controversial outcomes regarding the bond strength. Al saffan et al. reported the highest bond strength with CHX compared to using methylene blue as an aPDT agent or Nd:YAG laser (Al Saffan et al., 2021). Also, in another study conducted by Alshahrani et al., resin-modified glass ionomer cement bonded to CHX disinfected caries-affected dentin displayed the maximum shear bond strength, while methylene blue mediated aPDT had the lowest SBS. The results of LED application with curcumin and Er, Cr:YSGG laser irradiation were comparable to the CHX group (Alshahrani et al., 2020). These controversial reports necessitate more experiments on the adhesive bond strength of resin composites following different disinfection protocols.

Most of this assessed clinical studies recruited a laser with 630–660 nm wavelength with methylene blue or TB as a PS, some reporting a significant reduction in the count of *S. mutans* after treatment compared to the control. All in all, many *in-vitro* and clinical studies indicate the efficacy of aPDT in prevention and treatment of dental caries and increasing the bond strength between restoration and tooth structure; however, in order to obtain conclusive results, more clinical studies with standardized methodology are required. Considerable heterogeneity exists in irradiation protocols and study deigns which need to be considered and addressed in future studies.

## Applications of aPDT in the treatment of oral fungal infections

Candida species are natural members of a healthy microbial flora in the oral cavity and are in commensalism with other members of the microbial flora of mouth; however, they can become pathogenic and irritate the mucosa, especially when the host’s immune system is dysfunctional (Giannini and Shetty, 2011; Singh et al., 2014). Candidiasis is one of the most prevalent diseases in oral mucosa that is mainly caused by *Candida albicans* (*C. albicans*). This microorganism has also been proposed as the main pathogen isolated from the denture of the patients suffering from denture stomatitis (DS). Practicing an appropriate dental and prosthetic hygiene routine is necessary to prevent fungal infections and stomatitis; nevertheless, it is difficult for people with disabilities or hospitalized elderlies to effectively clean their mouth or disinfect their prostheses which can lead to oral infections and the use of antifungals (Papadiochou and Polyzois, 2018; Khadka et al., 2020).

As the known situation with antibiotics against bacteria, there has been increasing resistance to antifungal treatments due to the widespread use of these drugs, and the conduction of inadequate therapies in time or doses, is making the conventional therapies less and less effective. In addition, these medications have a limited range of action, and they can be toxic (Morio et al., 2017; Rodríguez-Cerdeira et al., 2021). Thus, other therapeutic approaches have been investigated against oral fungal infections in recent years, e.g., using oregano oil, tea tree oil, ozone therapy, nanoparticles, and light therapy (Ninomiya et al., 2013; Szweda et al., 2015; Maciel et al., 2016; Bhat et al., 2018; Monzillo et al., 2020; Pérez-Laguna et al., 2021). Many studies have introduced aPDT as a promising approach instead of the conventional antifungal treatments in managing oral candidiasis and denture stomatitis (Kato et al., 2013; Azizi et al., 2016; Janeth Rimachi Hidalgo et al., 2019; Alves et al., 2020). Several systematic reviews have been published on the effects of utilizing aPDT indicating the ever-increasing potential of aPDT as an effective antifungal therapy, but the consistency between the different study protocols is low. A summary of some of these studies can be found in Table 5.

**Table 5:** aPDT treatment in patients with fungal lesions.

Author/year	Study design	Study groups	Investigated pathology	Photosensitizer type/concentration	Light type and irradiation parameters	Microorganism	Follow-up periods	Outcomes
Afroozi et al. (2019)	Randomized clinical trial	Test: PDT + nystatin Control: nystatin	Denture stomatitis	Indocyanine green, 1 mg/ml	Diode laser: 810 nm, 56 J/cm^2^, 30 s Two sessions	*Candida* spp.	2 months	aPDT showed a significantly higher reduction in the number of candida CFU.
Alrabiah et al. (2019)	Randomized clinical trial	Test: aPDT Control: topical nystatin	Denture stomatitis	Methylene blue, 450 μg/ml	Diode laser: 660 nm, 100 mW, 28 J/cm^2^ Eight sessions	*C. albicans C. tropicalis C. glabrata*	1 and 2 months	Both treatments significantly reduced the number of *C. albicans*; however, the difference between them was not significant.
Alves et al. (2020)	Randomized clinical trial	Test: aPDT Control: topical nystatin	Denture stomatitis	Photodithazine, 200 mg/l	LED: 660 nm, 50, 240 mW/cm^2^, 50 J/cm^2^, 4, 17 min Six sessions	*C. albicans C. tropicalis C. glabrata*	15, 30, and 45 days	aPDT was more effective in the reduction of Candida spp. than NYS. Both groups showed recurrence.
de Senna et al. (2018)	Randomized clinical trial	Test: aPDT Control: oral miconazole gel	Denture stomatitis	Methylene blue, 450 μg/ml	Diode laser: 660 nm, 100 mW, 28 J/cm^2^ Eight sessions	*C. albicans C. tropicalis C. glabrata*	7, 15, and 30 days	aPDT was more effective in alleviating the inflammation after 15 days of treatment but the difference was not significant after 30 days.
Maciel et al. (2016)	Randomized clinical trial	Test: aPDT + LLLT Control: oral miconazole gel	Denture stomatitis	Methylene blue, 0.01%	Diode laser: 660 nm, 100 mW/cm^2^, 1 J/cm^2^, 10 s One session aPDT + four sessions LLLT	*Candida* spp.	1 month	The recurrence rate was lower in the patients treated with miconazole.
Mima et al. (2012)	Randomized clinical trial	Test: aPDT Control: topical nystatin	Denture stomatitis	Photogem (hematoporphyrin derivative), 500 mg/l	LED: 455 nm, 24 mW/cm^2^, 37.5, 122 J/cm^2^, 20, 26 min Six sessions	*C. albicans C. tropicalis C. glabrata*	1, 2, and 3 months	Both of the control and test groups resulted in clinical success rates of 53 and 45%. No difference was observed between the effectiveness of aPDT and NYS in the treatment of DS.
Scwingel et al. (2012)	Randomized clinical trial	Test 1: LLLT Test 2: aPDT Control: Fluconazole	Oral candidiasis	Methylene blue, 450 μg/ml	NR, 660 nm, 30 mW, 7.5 J/cm^2^, 10 s/point Single session	*Candida* spp.	0, 3, 7, and 30 days	aPDT eliminated the *Candida* spp. colonies and prevented the recurrence.

NR, not reported; nm, nanometers; μM, micrometer; mW, milliwatts; s, seconds; PS, photosensitizer; DS, Denture stomatitis: LLLT, Low-level laser therapy; NYS, nystatin.

Yeasts are less sensitive to PS agents compared to bacteria because of their size and the presence of a thick cell wall, so only a few PSs and light sources can be used in order to successfully eliminate them (Sousa et al., 2016; Wiench et al., 2019). The thin channels in the cell wall prevent the PS from passing through the wall; therefore, cationic PS and more extended contacts with the wall than the gram-negative bacteria are needed to ensure the yeast’s death. The Main examined PSs for fungal inactivation in the literature are methylene blue, toluidine blue O, indocyanine green, and Photogem®. Wiench et al. systematically reviewed toluidine blue O mediated aPDT on *Candida* spp. (Wiench et al., 2021). Analyzing the 21 included studies showed the following results: In the experiments with planktonic cells, one study showed complete annihilation of *C. albicans*, and others were partially effective. Also, one study did not show any significant difference (Merigo et al., 2019). Experiments conducted on the yeast biofilm indicated not complete but statically significant reduction in the cell number and growth. Reduced adhesion of *C. albicans* to epithelial cells and inhibited penetration ability into the epithelium have been reported (Dai et al., 2011; Sherwani et al., 2015).

Based on the findings of a systematic review and meta-analysis of 5 articles by Vila-Nova et al., the aPDT can be beneficial for reducing the colony-forming units on the palate and denture, but the conventional antifungal treatment revealed better performance after 15 and 30 days probably because the drugs can penetrate the pores in the denture and remain in them (Vila-Nova et al., 2022). Boltes Cecatto et al. also did a systematic review on methylene blue-mediated aPDT in human clinical studies (Boltes Cecatto et al., 2020). Of the five selected studies, two were on onychomycosis, one about oral candidiasis in HIV patients, and two about infected diabetic feet. In the oral candidiasis article, three approaches were used: Conventional antifungal therapy, phototherapy, and photodynamic therapy by methylene blue with the same irradiation parameters (660 nm, 30 mW, 7.5 J/cm^2^). While photobiomodulation did not show any *candida* spp. reduction, both aPDT and conventional medication decreased the number of cells. Complete elimination of fungus colonies without any reoccurrence was observed in aPDT group, but medication did not prevent the return of candidiasis (Scwingel et al., 2012).

Du et al. practiced aPDT using methylene blue plus potassium iodide (KI) in adult patients suffering from acquired immune deficiency syndrome (AIDS). They divided the patients into two groups with 400 and 600 μM methylene blue concentration and LED light with 633 nm wavelength and energy density of 37.29 J/cm^2^, however, there were no evidence of randomization in the study protocol. According to their results, although there was no significant difference between the 400 and 600 μM methylene blue concentrations, both protocols alleviated the clinical symptoms between 50% and 75% and reduced the number of the fungal cells and control opportunistic fungus. One or two aPDT sessions did not significantly affect the biofilm formation capacity of *C. albicans* (Ajmal, 2021). In another study, effectiveness of total mouth aPDT in individuals with AIDS was evaluated using 50 μg/ml porphyrin as PS and 660 nm LED. Though the treatment was able to reduce the general count of microorganisms in the oral cavity, the reduction in the number of Log10 CFU/ml of *Candida* spp. was not significant (da Silva et al., 2022). Wiench et al. introduced aPDT using toluidine blue O and a 635 nm diode laser with the energy density of 24 J/cm^2^, 400 mW power, and 30 s irradiation time as a possible therapeutic approach in future clinical studies (Wiench et al., 2019).

Dias et al. reported that using aPDT (660 nm, 18 J/cm^2^, 34 mW/cm^2^) successively three times using Photodithazine® 25 mg/L could completely inactivate the *C. albicans* in planktonic cultures; however, to prevent the re-cultivation of the cells in the biofilm model 5 sessions of aPDT was required (Bhat et al., 2018). Biofilm makes *C. albicans* more resistant to antimicrobial photodynamic therapy agents by creating extra protective layers. In compound biofilms containing more than one species, there is a higher possibility of developing a more resistant polymeric extracellular matrix as a result of a mutual coaggregation of the fungal species hampering the inactivation process (Falsetta et al., 2014; Kim et al., 2018). In the mentioned research, fluconazole was presented as an agent that can potentiate the aPDT regardless of the presence of biofilm. To destroy a biofilm model, the concentration of the PS must be 100 times greater than the one used in a planktonic model (Rodríguez-Cerdeira et al., 2021).

A randomized controlled trial by Alrabiah et al. compared the efficacy of aPDT and local nystatin therapy for denture stomatitis treatment. Thirty-six individuals were divided to two groups, one utilizing GaAlAs diode laser (660 nm, 100 mW, 28 J/cm^2^) and methylene blue (450 μg/ml) as PS. The other group used topical nystatin oral suspension of 100,000 IU four times a day for 15 days. They stated that the recorded CFU/ml values were not different between the groups throughout the study, and aPDT was as effective as nystatin for treating denture stomatitis (Alrabiah et al., 2019). This result was also confirmed by Mima et al. examining Photogem 500 mg/L irradiated by LED in a clinical trial comparing the effects of conventional antifungal therapy with aPDT (Mima et al., 2012). Afroozi et al. investigated the effect of indocyanine green-mediated aPDT (1 mg/ml) in combination with nystatin (100,000 U) in the management of denture stomatitis in comparison with the conventional nystatin therapy. Sixty-six patients were assigned into two groups and received nystatin mouthwash three times a day for 15 days, but the aPDT group also got laser irradiation (810 nm, 56 J/cm^2^) twice a day once a week. Evaluations showed that the mean reduction in the number of *Candida* spp. was markedly higher in the aPDT + nystatin group suggesting it as an alternative to the currently available antifungal therapies (Afroozi et al., 2019).

Oral mucositis is a common side effect, of chemotherapy and radiotherapy in the process of cancer treatment, which makes the patient susceptible to infections caused by opportunistic microorganisms such as *Candida* spp. (Trotti et al., 2003). Several protocols have been suggested to alleviate the pain and the inflammation like promoting oral hygiene, using antibiotics, analgesics, growth factors, anti-inflammatory agents, photobiomodulation, and antimicrobial photodynamic therapy (Fekrazad and Chiniforush, 2014). Andrade et al. compared the efficacy of photobiomodulation and Curcumin mediated aPDT as an adjuvant therapy of oral mucositis in oncologic patients using a 450 nm LED with 20.1 J/cm^2^ energy density. Their results illustrated a lower number of CFUs and lower degree of mucositis in the aPDT group on 21th and 30th days of follow-up compared to the control group. There was no statically significant difference between the aPDT and PBM groups indicating that they are both effective therapies in the management of oral mucositis resulted from chemotherapy and radiotherapy (de Cássia Dias Viana Andrade et al., 2022). As been said, one of the primary factors affecting the results of photodynamic therapy is the light parameter, including the wavelength, energy density, power density, and irradiation time. Most of the reviewed articles used a wavelength ranging between 630 and 660 nm and an incubation time of 5 to 20 min. It is essential to use a light source that induces the most absorption of PS in the cells, which also depends on the type and concentration of the PS solution (D'Ilario and Martinelli, 2006). Photodithazine derivatives were the most used sensitizing agents that displayed no clinical adverse effects and are considered safe substances to be used in treating superficial fungal infections in a controlled procedure. Of all the discussed pieces of research, most of them reported an improvement in the clinical features of the evaluated fungal disease or a reduction in the CFUs of samples.

Even though the effectiveness of aPDT can be supported to some extent as an adjunct fungal therapy, the quality of many of them is not satisfactory; therefore, more clinical trials are needed in order to determine the ideal amount of efficacious factors and the safety of the approach (Boltes Cecatto et al., 2020).

We were able to identify several studies have evaluated the effect of aPDT on oral fungi such as *C. albicans* compared with treatments with Nystatin as an antifungal reporting it to be able to result in higher reduction in the aPDT treated groups. These studies have mostly used Methylene blue as a PS and wavelengths of 660 nm either with diode lasers or LED devices were used. ICG was used in one study with a wavelength of 810 nm that is suitable for its activation and reported higher reduction of candida CFU in patients with denture stomatitis (Afroozi et al., 2019).

Photogem which is a hematoporphyrin derivative mostly used in photodynamic drug therapy of malignant tumors, was used in one study. The PS was activated with 455 nm LED and resulted in clinical success rate of 53% compared to 45% in the control group that used topical Nystatin as their treatment. They concluded that aPDT can be almost as equally effective in treatment of denture stomatitis as Nystatin (Mima et al. 2012).

## Applications of aPDT in the treatment of oral viral infections

aPDT has been introduced as a potential treatment against the viral diseases when used as an adjunctive treatment alongside the antiviral medications with promising results by the recently published RCTs (Ajmal, 2021; Ramalho et al., 2021; Shetty et al., 2022; Table 6). These studies have all used methylene blue as a PS activated with a red wavelength of 660 nm. Viral infections usually have manifestations such as blister or ulcers in the oral cavity that can be very irritating for patients. They have also been considered to play a role in periodontal disease and some oral cancers (Healy and Moran, 2019; Puletic et al., 2020; Tsuchida, 2020; Sarkar et al., 2021; Hajishengallis, 2022). The conventional treatment for viral infections is the use of antiviral medications such as acyclovir which is a synthetic acyclic purine-nucleoside analog in the case of herpes simplex virus (HSV) infections as the etiology of herpetic gingivostomatitis (Whitley and Roizman, 2001). However, these infections are prone to get resistant against antiviral drugs after a long-term topical, oral and intravenous use which reduces the effect of antiviral medications over time. Also, the oral or intravenous application is only for the severe or high-rate recurrence of viral infections (Frobert et al., 2014). The mechanisms leading to the viral infection resistance have been explained by the mutations occurred in the virus genes responsible for encoding thymidine kinase, generating thymidine-kinase-deficient mutants which cannot phosphorylate acyclovir (Ramalho et al., 2021). It has been shown that aPDT can photo-inactivate both DNA and RNA viruses. Further, photo-inactivation of HSV by methylene blue as a cationic charged PS has shown promising outcomes, especially that the viral infections are unable to become resistant against aPDT (de Paula Eduardo et al., 2014).

**Table 6:** aPDT treatment in patients with oral viral infections.

Author/year	Study design	Study groups	Photosensitizer type/concentration	Light type and irradiation parameters	Microorganism	Follow-up periods	Outcomes
Ajmal (2021)	Randomized clinical trial	Test 1: Acyclovir Test 2: aPDT Test 3: aPDT + Acyclovir	Methylene blue, 0.005%	Diode laser: 660 nm, 150 mW, 300 J/cm^2^, 30 s single session	Herpes simplex virus type 1	0, 2, and 4 weeks; 3 and 6 months	Group aPDT + Acyclovir showed the most significant reduction in the quantified HSV-1, pain scores, and reported levels of IL-6 and TNF-α compared to other groups. No difference was observed in terms of pain scores among groups.
Ramalho et al. (2021)	Randomized clinical trial	Test 1: aPDT Test 2: Acyclovir Test 3: aPDT + Acyclovir	Methylene blue, 0.005%	Low-power laser: 660 nm, 40 mW, 120 J/cm^2^, 120 s per point single session	Herpes Simplex Virus Type 1	7 days	On day 1, the AC group showed less wound size reduction and higher edema compared to the AC-PDT group. No significant differences were observed in the size of the lesion between groups from day 2. aPDT and Acyclovir showed no significant difference regarding healing time, edema and pain.
Vellappally et al. (2022)	Randomized clinical trial	Test 1: topical anti-viral therapy Test 2: aPDT Test 3: aPDT + topical anti-viral therapy	Methylene blue, 0.005%	640 nm, 4 J, 300 J/cm^2^, 150 mW, 0.025 cm^2^, and 30–40 s for each lesion			

NR, not reported; nm, nanometers; μM, micrometer; mW, milliwatts; s, seconds.

There is a lack of robust RCTs investigating the treatment of oral viral infections with aPDT as the main or adjunct treatments. Vellappally et al. and Ajmal investigated the effect of aPDT (methylene blue 0.005% as PS) for the treatment of herpes labialis in adolescent population. Both demonstrated that using aPDT in adjunct with acyclovir reduced the pain scores as a parameter mostly important for patients in addition to the molecular level parameters including quantified HSV-1, IL-6, and TNF-α (Ajmal, 2021; Shetty et al., 2022). In another RCT by Ramalho et al., with similar methodology on the adult population, application of adjunctive aPDT yielded no substantial difference compared to the acyclovir in terms of lesion healing time, edema and pain. Furthermore, no side effects were reported in the groups containing aPDT treatment by the participants (Ramalho et al., 2021). Given the promising but scarce evidence concerning the effect of aPDT in the treatment of oral viral infection, it is suggested to conduct more RCTs to investigate the efficiency of this method to treat oral viral conditions associated with viruses such as Epstein–Barr virus, cytomegalovirus, human herpesviruses and herpes zoster and even SARS-CoV-2, in healthy and/or immunocompromised patients (Patel and Woolley, 2021; Thakkar et al., 2022).

## Future perspectives

Although the efficacy of aPDT is approved by many studies, there are some downsides of using it as an adjuvant in clinical therapies. Poor target selection, an uncontrollable manner in drug releasing, poor water solubility, high cost of the PSs, and low oxygen concentrations in deeper parts of the targeted tissues are some factors limiting PS usage in future clinical trials (Yin et al., 2015). In order to deal with these factors and improve their antimicrobial performance, researchers have been working on novel approaches such as utilizing nanoparticles (NPs) as vehicles to transfer hydrophobic PS into the microorganisms, using nanomaterials with similar properties as PSs or conjugating PSs with monoclonal antibodies that possess better targeting characteristics than the PS alone (Schmitt and Juillerat-Jeanneret, 2012). With the development of various nanomaterials, it is of great importance to consider the potential long-term toxicity of not only the tested NPs but also the safe drugs that have not shown any toxic effects in previous studies with short-term follow-ups since the long-term adverse manifestations may be observed after a long interval (Qi et al., 2019). Further, pigmenting the patient’s teeth and gums is another issue with PSs that can be minimized by using nanocarriers that protect the treated area from being colored (Silvestre et al., 2021).

Studies on novel designs of PSs evaluating their antimicrobial effects on *S. mutans* have shown more effective bacterial reduction and PSs’ characteristics, when conjugated tolouidine blue O with silver nanoparticles (AgNPs) and nanocarriers containing Graphene Oxide-Carnosine/Hydroxyapatite loaded with indocyanine green has been used (Misba et al., 2016; Gholibegloo et al., 2018). Also, photoactivation-independent PSs like Rose bengal-functionalized chitosan NPs (CSRBnp) can significantly reduce the inflammatory marker expressed from macrophages and efficiently inactivate the endotoxins and lipopolysaccharides (Shrestha et al., 2015).

Novel designs of PSs such as Nano-Graphene oxide conjugated with indocyanine green are able to increase the bactericidal characteristics against biofilm formation of *Enterocucus faecalis* with lower effective concentrations than indocyanine green alone (Akbari et al., 2017). Also, new PS structure designing techniques, can make the previously not efficient PSs effective, like indocyanine green -loaded NPs covered with chitosan which is able to significantly reduce the viability and the load of *P. gingivalis* whereas indocyanine green alone could not affect the *P. gingivalis* due to its positive surface charge (Nagahara et al., 2013). *Porphyromonas gingivalis* is considered as a keystone pathogen and the major culprit not only in periodontal destruction but also in the relationship between periodontitis and systemic conditions such as diabetes, cardiovascular diseases (Liccardo et al., 2019), preterm low birth weight (Teshome and Yitayeh, 2016) and Alzheimer’s (Borsa et al., 2021; Ding et al., 2021). Therefore, a local and non-invasive method to effectively reduce and control the load inherent oral environment bacteria such as *P. gingivalis*, can be considered as promising modalities of overcoming its related adverse effects on end organ systemic diseases.

Considering the promising results of aPDT against viruses (Namvar et al., 2019), and recently emerged SARS-CoV-2, there is so much to exploit from this method in future studies specifically in the field of virology and dentistry. Also developing anticaries vaccines and modulating the oral microbiota to non-pathogen species is not impossible (Ghazi et al., 2021).

## Conclusion

Considering the effectiveness of aPDT against a wide range of micro-organisms and its promising results demonstrated in some clinical studies; it is highly suggested that further *in-vivo* studies and clinical trials be conducted with more detailed and homogenous study designs to optimize irradiation protocols and wavelengths suitable for PS activation. Systematic reviews on the different clinical application areas with metanalysis of the results are needed to pave the way toward evidence-based application of aPDT in dentistry. Moreover, novel methods of PS structure design improved by carriers and adjuvants to enhance the current conventional therapies’ safety, efficacy, targeting and cost-effectiveness can help clinicians reach the desired therapeutic goals. Patient satisfaction and safety/adverse effects are also an important aspect that needs to be considered and evaluated in future clinical studies.

## Author contributions

MH: methodology, investigation, data curation, and writing—original draft. SS and MJ: investigation, data curation, and writing—review and editing. LG: supervision and writing—review and editing. RF: conceptualization, validation, supervision, project administration, and review and editing. All authors contributed to the article and approved the submitted version.

## Conflict of interest

The authors declare that the research was conducted in the absence of any commercial or financial relationships that could be construed as a potential conflict of interest.

## Publisher’s note

All claims expressed in this article are solely those of the authors and do not necessarily represent those of their affiliated organizations, or those of the publisher, the editors and the reviewers. Any product that may be evaluated in this article, or claim that may be made by its manufacturer, is not guaranteed or endorsed by the publisher.
