# Supplementary material for: Clinical applications of antimicrobial photodynamic therapy in dentistry
Source: Front Microbiol. 2023 Jan 5;13:1020995. doi: 10.3389/fmicb.2022.1020995 (PMC9850114; doi:10.3389/fmicb.2022.1020995)
Supplement: Supplementary file 1 [file Data_Sheet_1.docx]

**Search strategy and keyword**

**PubMed**

((((((((((((antimicrobial PDT) OR (antimicrobial PDT (aPDT))) OR (antimicrobial photo-dynamic therapy (aPDT))) OR (antimicrobial photo-dynamic)) OR (antimicrobial photodynamic inactivation therapy)) OR (antimicrobial photodynamic therapy)) OR (antimicrobial photodynamic therapy (aPDT))) OR (antimicrobial photodynamic treatment)) OR (aPDT (antimicrobial photodynamic therapy))) OR (aPDT)) OR (photodynamic antimicrobial therapy)) AND (dent* OR oral OR mouth OR tooth OR periodontitis OR peri-implantitis OR implant OR endodontic* OR caries OR carious OR virus* OR viral OR fungus OR fungi OR fungal OR gingivitis OR gingiva*)) AND ((((((controlled trial[Title/Abstract]) OR (randomised controlled study[Title/Abstract])) OR (randomised controlled trial[Title/Abstract])) OR ("randomized controlled"[Title/Abstract])) OR (Clinical Trial[Title/Abstract])) OR (RCT[Title/Abstract]))

**Web of Science**

---------------------------------------------------------------#1--------------------------------------------------------------------------
TS=("antimicrobial PDT" OR "antimicrobial PDT (aPDT)" OR "antimicrobial photo-dynamic therapy (aPDT)" OR "antimicrobial photo-dynamic" OR "antimicrobial photodynamic inactivation therapy" OR "antimicrobial photodynamic therapy" OR "antimicrobial photodynamic therapy (aPDT)" OR "antimicrobial photodynamic treatment" OR "aPDT (antimicrobial photodynamic therapy)" OR "aPDT" OR "photodynamic antimicrobial therapy")

---------------------------------------------------------------#2--------------------------------------------------------------------------

TS=("dent*" OR "oral" OR "tooth" OR "mouth " OR "periodontitis " OR "peri-implantitis " OR "implant " OR "endodontic*" OR "caries" OR "carious" OR "virus*" OR "viral" OR "fungus*" OR "fungi" OR "fungal" OR "gingivitis" OR "gingiva*")

---------------------------------------------------------------#3--------------------------------------------------------------------------

TS=("controlled trial" OR "randomised controlled study" OR "randomised controlled trial" OR "randomized controlled" OR "Clinical Trial" OR "RCT " )

---------------------------------------------------------------#4--------------------------------------------------------------------------

#3 AND #2 AND #1

**Scopus**

( TITLE-ABS-KEY ( "antimicrobial PDT" OR "antimicrobial PDT (aPDT)" OR "antimicrobial photo-dynamic therapy (aPDT)" OR "antimicrobial photo-dynamic" OR "antimicrobial photodynamic inactivation therapy" OR "antimicrobial photodynamic therapy" OR "antimicrobial photodynamic therapy (aPDT)" OR "antimicrobial photodynamic treatment" OR "aPDT (antimicrobial photodynamic therapy" OR "aPDT" OR "photodynamic antimicrobial therapy") ) AND ( TITLE-ABS-KEY ( "dent*" OR "oral" OR "tooth" OR "mouth " OR "periodontitis " OR "peri-implantitis " OR "implant " OR "endodontic" OR "caries" OR "carious" OR "virus*" OR "viral" OR "fungus*" OR "fungi" OR "fungal" OR "gingivitis" OR "gingiva*" ) ) AND ( TITLE-ABS-KEY ("controlled trial" OR "randomized controlled study" OR "randomised controlled trial" OR "randomized controlled" OR "Clinical Trial" OR "RCT ") )

**Embase**

('antimicrobial photodynamic therapy'/exp OR 'antimicrobial photodynamic therapy' OR 'antimicrobial pdt':ti,ab OR 'antimicrobial pdt (apdt)':ti,ab OR 'antimicrobial photo-dynamic therapy (apdt)':ti,ab OR 'antimicrobial photo-dynamic':ti,ab OR 'antimicrobial photodynamic therapy':ti,ab OR 'antimicrobial photodynamic therapy (apdt)':ti,ab OR 'antimicrobial photodynamic treatment':ti,ab OR 'apdt (antimicrobial photodynamic therapy':ti,ab OR 'apdt':ti,ab OR 'photodynamic antimicrobial therapy':ti,ab) AND ('dent*':ti,ab OR 'oral':ti,ab OR 'tooth':ti,ab OR 'mouth':ti,ab OR 'periodontitis':ti,ab OR 'peri-implantitis':ti,ab OR 'implant':ti,ab OR 'endodontic':ti,ab OR 'caries':ti,ab OR 'carious':ti,ab OR 'virus*':ti,ab OR 'viral':ti,ab OR 'fungus*':ti,ab OR 'fungi':ti,ab OR 'fungal':ti,ab OR 'gingivitis':ti,ab OR 'gingiva*':ti,ab) AND ('randomized controlled trial'/exp OR 'randomized controlled trial' OR 'controlled trial'/exp OR 'controlled trial' OR 'randomised controlled study'/exp OR 'randomised controlled study' OR 'randomised controlled trial':ti,ab OR 'randomized controlled':ti,ab OR 'clinical trial':ti,ab OR 'rct':ti,ab)

**PRISMA flowchart**

Records identified from:

Databases (n = 1042)

PubMed: 190

Embase: 448

Scopus: 242

Web of Science: 162

Records removed *before screening*:

Duplicate records removed (n = 340 )

Records screened

(n = 702)

Records excluded

(n =453)

Reports assessed for eligibility

(n =249)

Studies included in review

(n =89)

**Identification of studies via databases and registers**

**Identification**

**Screening**

**Included**
